# Symbiont-Driven Male Mating Success in the Neotropical *Drosophila paulistorum* Superspecies

**DOI:** 10.1007/s10519-018-9937-8

**Published:** 2018-11-19

**Authors:** Daniela I. Schneider, Lee Ehrman, Tobias Engl, Martin Kaltenpoth, Aurélie Hua-Van, Arnaud Le Rouzic, Wolfgang J. Miller

**Affiliations:** 1 Department of Cell and Developmental Biology, Center of Anatomy and Cell Biology, Medical University of Vienna, Schwarzspanierstrasse 17, 1090 Vienna, Austria; 2 Natural Sciences, State University of New York, Purchase College, Purchase, NY USA; 3 Department for Evolutionary Ecology, Institute for Organismic and Molecular Evolution, Johannes Gutenberg-Universität, Mainz, Germany; 4 Évolution, Génomes, Comportement, Écologie, CNRS, Institut de Recherche pour le Développement, Université Paris-Sud, Université Paris-Saclay, 91198 Gif-sur-Yvette, France; 5 Present Address: Department of Epidemiology of Microbial Diseases, Yale School of Public Health, New Haven, CT 06511 USA

**Keywords:** *Wolbachia*, *Drosophila*, Reproductive isolation, Premating isolation, Pheromones

## Abstract

Microbial symbionts are ubiquitous associates of living organisms but their role in mediating reproductive isolation (RI) remains controversial. We addressed this knowledge gap by employing the *Drosophila paulistorum*-*Wolbachia* model system. Semispecies in the *D. paulistorum* species complex exhibit strong RI between each other and knockdown of obligate mutualistic *Wolbachia* bacteria in female *D. paulistorum* flies triggers loss of assortative mating behavior against males carrying incompatible *Wolbachia* strains. Here we set out to determine whether de novo RI can be introduced by *Wolbachia*-knockdown in *D. paulistorum* males. We show that *Wolbachia*-knockdown *D. paulistorum* males (i) are rejected as mates by wild type females, (ii) express altered sexual pheromone profiles, and (iii) are devoid of the endosymbiont in pheromone producing cells. Our findings suggest that changes in *Wolbachia* titer and tissue tropism can induce de novo premating isolation by directly or indirectly modulating sexual behavior of their native *D. paulistorum* hosts.

**Electronic supplementary material:**

The online version of this article (10.1007/s10519-018-9937-8) contains supplementary material, which is available to authorized users.

## Introduction

Potential impacts of microbial symbionts as drivers of host speciation have been discussed frequently (Bordenstein et al. 2001; Jaenike et al. 2006; Telschow et al. 2007, 2014; Brucker and Bordenstein 2012; Gebiola et al. 2016), but their broader evolutionary significance fostering speciation remains controversial (Brucker and Bordenstein 2013, 2014; Chandler and Turelli 2014; Turelli et al. 2014; Najarro et al. 2015; Leftwich et al. 2017), and standard models of speciation commonly disregard such impacts (Coyne and Orr 2004; Brucker and Bordenstein 2012; Miller and Schneider 2012). Several theoretical models have nominated the maternally transmitted reproductive tract endosymbiont *Wolbachia* as a candidate worthy of consideration (Telschow et al. 2007, 2014). The α-proteobacterium *Wolbachia* is arguably the most prevalent intracellular invertebrate infection on the planet, infecting as many as 40% of all terrestrial arthropods (Zug and Hammerstein 2012). *Wolbachia* have attracted attention as participants in arthropod symbioses because they successfully manipulate host biology in manifold ways, ranging from reproductive parasitism like cytoplasmic incompatibility (CI), male killing, feminization and parthenogenesis to nutritional supplementation, fecundity and protection from pathogens and parasites (Werren et al. 2008; Teixeira et al. 2008; Brownlie et al. 2009; Fast et al. 2011; Moriyama et al. 2015). Although *Wolbachia* are mainly found as facultative endosymbionts in most insect hosts (Martinez et al. 2015 and references therein), they also can evolve fixed obligate associations such as in the parasitoid wasp *Asobara* (Dedeine et al. 2001), bedbugs (Hosokawa et al. 2010), or neotropical fruit flies belonging to the *Drosophila paulistorum* species complex (Miller et al. 2010).

In the latter case, this superspecies is under incipient speciation in the wild and consists of six semispecies (Dobzhansky and Spassky 1959), named Amazonian (AM), Andean Brazilian (AB), Centroamerican (CA) Interior (IN), Orinocan (OR) and Transitional (TR), expressing strong RI at the pre- and postzygotic level in inter-semispecies crosses (reviewed in Ehrman and Powell 1982). As recently found they all carry different loads of obligate mutualistic but distinctive *Wolbachia* strains (Miller et al. 2010), which can cause strong cytoplasmic incompatibilities (embryonic mortality) plus complete hybrid male sterilities in reciprocal crosses between different semispecies in the laboratory. Because *Wolbachia* are obligate endosymbionts in this system, no uninfected flies can be generated (Ehrman 1968; Kernaghan and Ehrman 1970; Miller et al. 2010) to test for classic bidirectional CI as feasible in facultative symbioses such as in *Culex pipiens* mosquitos (reviewed in Werren et al. 2008). The association between the obligate *Wolbachia* symbiont (earlier designated *Mycoplasma-like-organisms, MLO*s; reviewed in Ehrman and Powell 1982) and the induction of postmating isolation, however, is based on earlier and recent findings that hybrid lethality and male sterility are partly reversible upon mild antibiotic or heat treatments of the parents before mating (Ehrman 1968; Kernaghan and Ehrman 1970; Miller & Schneider, unpublished).

In two earlier publications, Dobzhansky and collaborators (Dobzhansky and Pavlovsky 1966, 1971) observed the spontaneous evolution of de novo postmating isolation as some lines, which were originally fully compatible with the Orinocan reference strain O11, were no longer compatible with this semispecies or any other semispecies of *D. paulistorum*. This resulted in occurrence of high embryonic F_1_ mortality and complete male hybrid sterility, presumably because of drift and isolation in these lines in the laboratory. However, the role of endosymbionts on the formation of premating mechanisms has been less studied in the past (Dobzhansky and Pavlovsky 1966, 1971; Miller et al. 2010). Importantly, in facultative symbioses, where *Wolbachia* has not reached fixation in their natural hosts as in *D. melanogaster* or *D. yakuba* group species, the authors did not detect any *Wolbachia* effect on premating isolation (Sharon et al. 2010; Arbuthnott et al. 2016; Cooper et al. 2017).

**Fig. 1:**
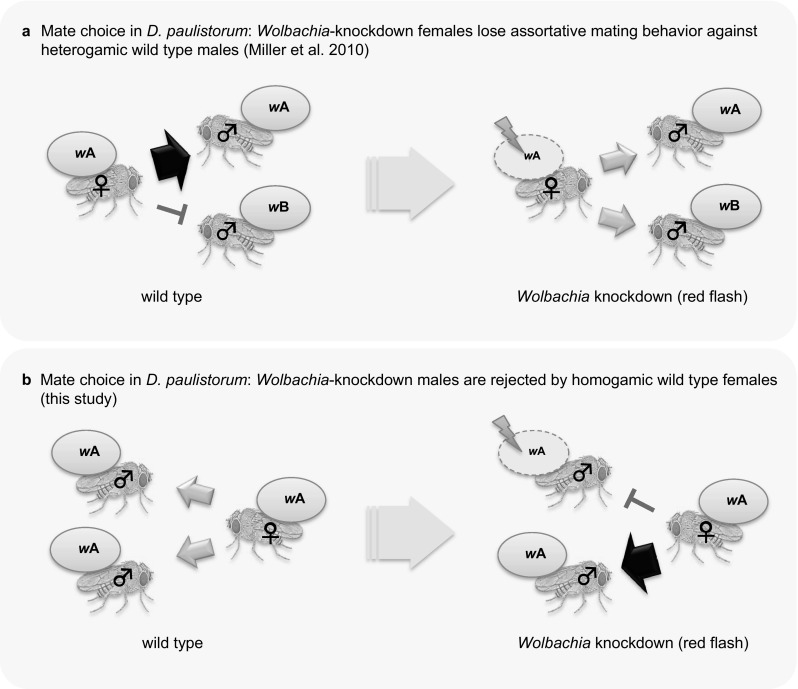
Impact of *Wolbachia* on mate choice in *D. paulistorum*. **a** Mate choice in *D. paulistorum: Wolbachia*-knockdown females lose assortative mating behavior against heterogamic wild type males. Left: wild type females (blue) infected with *Wolbachia* A (*w*A) express strong assortative mating behavior (red block) against males of a different semispecies (red) carrying a different, incompatible *Wolbachia* (*w*B). Right: *Wolbachia* kd females (symbolized with red flash) with reduced titers of *w*A lose assortative mating behavior (grey arrows) and hence mate randomly with males from same (blue) but also from different/incompatible (red) semispecies. Female knockdown phenotype: loss of assortative mating behavior. Data taken from (Miller et al. 2010). **b** Mate choice in *D. paulistorum: Wolbachia*-knockdown males are rejected by homogamic wildtype females. Left: wild type males are accepted randomly (grey arrows) for mating by females of the same semispecies (all blue), carrying the same/compatible *Wolbachia* (both *w*A). Right: *Wolbachia* kd males (indicated by red flash) with reduced titers of *w*A are rejected as mate partners (red block) from wild type females of the same semispecies. Thickness of arrows indicates strength of mate choice between partners: thick black arrow = high mate preference, red block = assortative mating, and grey medium arrow = lack of mate choice, i.e., random mating. *kd* knockdown. (Color figure online)

This current study is based on the recent observation that *D. paulistorum* semispecies show strong premating isolation through female mate choice for intra-semispecific (homogamic) males (Fig. 1A, left panel). Such behavior is lost upon *Wolbachia-*knockdown in females, i.e., significant titer reduction but not clearance, resulting in random mating between *per se* incompatible, heterogamic mates (Miller et al. 2010; Fig. 1A, right panel). Knockdown instead of clearance is performed because *Wolbachia* is a fixed obligate mutualist in *D. paulistorum*, providing vital but still undetermined functions for its native hosts (Miller et al. 2010). More recently, we could demonstrate selective neurotropism (the affinity to nervous tissue) of native *D. paulistorum Wolbachia* to defined female and male brain regions, known as functionally important for mating behavior and memory (Strunov et al. 2017). In addition to governing assortative mating behavior of *D. paulistorum* females, we speculate that perturbations of the *Wolbachia-Drosophila* homeostasis in males might induce assortative mating behavior between *per se* compatible, homogamic mates, at least under experimental conditions (Fig. 1B). Finally we speculate that under certain conditions like obligate mutualism and tropism of the endosymbiont to host organs associated with sexual behavior, *Wolbachia* can act as an influential and dynamic factor for modulating sexual behavior, which, because of their sensitivity to exogenous factors such as stress and antibiotics (Reynolds and Hoffmann 2002; Mouton et al. 2006; Weeks et al. 2007), plus their high innate mutability (Chrostek et al. 2013; Schneider et al. 2013; Newton and Sheehan 2014), can possibly initiate the process of sexual isolation, at least under experimental laboratory conditions. Their potential impact on speciation in nature, however, remains elusive.

The key components of premating sexual isolation mechanisms are visual, acoustic, and chemical courtship signals. In many insects, sexual pheromones play an important role as olfactory signals influencing mate choice (Blomquist and Bagnères 2010; Chung and Carroll 2015; Dembeck et al. 2015). In *D. paulistorum*, males from the six semispecies exhibit characteristic sexual pheromone profiles (same compounds but different quantities), including four male-specific long-chained ester compounds, which are recognized by females (Kim et al. 2004; Chao et al. 2010). In contrast to other *Drosophila* species such as *D. melanogaster*, no female-specific compounds are present in this system. Hence, the authors of previous studies (Kim et al. 2004; Chao et al. 2010) concluded that, based on chemical profiles, *D. paulistorum* females accept homogamic (compatible), but reject heterogamic (incompatible) males as mates (Chao et al. 2010). Forced mating between heterogamic mates carrying incompatible *Wolbachia* strains is highly detrimental for both host and symbionts by triggering high levels of embryonic lethality and complete male sterility among F_1_ hybrids (Ehrman 1968; Kernaghan and Ehrman 1970; Miller et al. 2010). Hence strong selection should act on both intimate partners to evolve or maintain such mechanisms for mate recognition.

Here we experimentally investigated whether bacterial symbionts are capable of initiating artificial de novo reproductive isolation (RI) by targeting host-derived olfactory cues. Therefore, we have (i) transiently knocked down obligate *Wolbachia* in *D. paulistorum* males, (ii) assayed their mating success in homogamic choice assays with wild type females, (iii) monitored their sexual pheromone profiles, and (iv) investigated the presence of *Wolbachia* in putative pheromone-producing cells.

## Methods

### Fly strains and generation of *Wolbachia*-knockdown (kd), axenic (et), and penicillin/streptomycin-treated (ps) flies

Reference strains for two semispecies from the *Drosophila paulistorum* superspecies were chosen for this study (Amazonian, A28 and Orinocan, O11; originally described in Burla et al. 1949; Dobzhansky and Pavlovsky 1966). The *Wolbachia* infection status (*w*Pau) of these *D. paulistorum* strains was previously described in Miller et al. 2010. In brief, A28 carries very low densities of the *Wolbachia w*PauAM strain, and O11 is infected with the high-titer *w*PauOR strain (Miller et al. 2010). *Wolbachia* positive controls for Polymerase chain reaction (PCR) assays were *D. willistoni* P98 and JS6.3, both lab strains carrying native high-titer *Wolbachia* (*w*Wil; Miller and Riegler 2006). The *D. melanogaster* H2-strain (*w*Mel; Miller and Riegler 2006) was used as an additional *Wolbachia* positive control. *Wolbachia* negative controls for PCR were *D. simulans* NouméaTC (Poinsot et al. 2000), *D. melanogaster* w^1118^ (BDSC, USA), and *D. willistoni* Willi3 (14030-0811.2 DSSC, USA). Flies were reared on Formula 4–24 *Drosophila* instant food (Carolina, USA) at 24–25 °C on a 12 h light–dark cycle.

*Wolbachia*-knockdown lines A28^kd^ and O11^kd^ were generated by mass-treating wild type flies with 0.2% (w/v) rifampicin (Duchefa, Netherlands) added to Formula 4–24 *Drosophila* instant food (Carolina, USA) over three sequential generations (T_1_–T_3_). The low dosage of the antibiotic is sublethal and allows for reduction of mutualistic *Wolbachia* in *D. paulistorum* (Miller et al. 2010). Offspring from T_3_ parents were then transferred onto antibiotic-free food for more than 10 generations under a mass-rearing regime designated as knockdown pools (A28^kd^, O11^kd^; Supporting Information Fig. S1). In parallel, F_2_^kd^ was used to initiate 8–10 isofemale lines per semispecies (A28^kd-i^, O11^kd-i^; Supporting Information Fig. S1). Biological assays were performed at different generations between F_4_^kd^ and F_13_^kd^ and their respective *Wolbachia* load was monitored by qPCR (see below). Gut flora restoration (gfr) lines were generated by feeding the native gut microbiome to knockdown flies as follows: A28^wt^ and O11^wt^ virgin females were kept on instant food for 2–3 days to collect feces. Flies were then removed and the corresponding knockdown (kd) flies were transferred onto the feces-containing food vials. After egg deposition adults were removed and emerging flies were consequently used as gfr lines. Axenic (‘gut microbe-free flies’) were generated by consequently washing freshly collected eggs from A28^wt^ and O11^wt^ with 70% ethanol for 5 min to surface-sterilize them. This treatment prevented larvae ingesting microbes from the outer layer of the eggshell when hatching. Collected eggs were then transferred into food vials and hatching F_1_ and F_2_ adults (A28^et^, O11^et^) were consequently used for mate choice assays. Penicillin/streptomycin-treated (ps) flies were generated by supplementing Formula 4-24 *Drosophila* instant food (Carolina, USA) with a 1:100 dilution of a 100× pen/strep stock solution (10,000 units/ml penicillin, 10,000 µg/ml streptomycin). A28^wt^ and O11^wt^ were kept on this food for at least 1 week to lay eggs and the hatching F_1_ (A28^ps^, O11^ps^) was consequently used for mate choice assays against the wt counterparts. Strains are reported in Table S1.

### Quantification of *w*Pau *Wolbachia*

DNA was extracted from a pool of ten 3-day old flies using Gentra Puregene chemistry (Qiagen, Germany). Concentrations of DNA were measured on a Nanodrop 2000 spectrophotometer. Consequently, diagnostic *Wolbachia*-PCR (quantitative real time PCR) was performed using the Wolbachia outer surface protein gene *wsp* (Yamada et al. 2007), and the *Wolbachia*-specific *16S* rRNA gene (16SW_RTf 5′-CCTGATCCAGCCATGCCGCAT-3′, 5′-16SW_RTr CGGCTGCTGGCACGGAGTTA-3′). The *wsp* primer set generates a 69 bp amplicon, and the *16S* rRNA set produces a 77 bp fragment. *Wolbachia* titers obtained with MxPro QPCR v4.10 Software (Agilent Technologies, USA) were normalized against *Drosophila* ribosomal protein *RPS17* (RPSmel_f 5′-CACTCCCAGGTGCGTGGTAT-3′, RPSwil_r 5′- GGAAACGGCGGGCACGTA-3′). A temperature profile of 95 °C for 3 s, 60 °C for 20 s, and 72 °C for 6 s was used for 45 cycles. All samples were run in duplicates on a Stratagene MxPro4000 cycler. Quantitative PCR with *wsp* was run to confirm results obtained with the *16S* rRNA primer set. Only results for the latter one are presented in the manuscript.

### Measuring sexual isolation via multiple mate choice assays

Mate choice assays between wild type, knockdown and control assays involved double blind direct multiple-mating observations carried out mornings at room temperature in daylight. Virgin flies were aged 2−3 days (females isolated from males), and half were marked *via* distal wing clips before running the choice assay. These marks were rotated (wing to wing and knockdown to wild type). Such minute abrasions have tested neutral regarding behavioral influences in this superspecies (Dobzhansky and Pavlovsky 1966; Leonard and Ehrman 1983). For each mate choice assay five replicas and 120 mating events (240 individual flies) were scored (12A ♀♀ + 12B ♀♀ + 12A ♂♂ + 12B ♂♂ differentiated by alternating wing clips; Supporting information Fig. S2a, b). Flies were placed (females first) in mating observation chambers (10 cm in diameter) without anesthetization and the following parameters were scored until all flies copulated in approximately 30–40 min: the time each mating took place (from start of observations; each copulation approximately lasted 15–17 min); its sequence among other *copulae* which occured; where in the chamber the mating pair was located; the kind of female involved; and the kind of male involved. Recording the location of each copula, even upside down, prevented scoring a copula more than once. Sexual isolation index (SII) was computed according to the following formula (Malogolowkin-Cohen et al. 1965).

SII = (nho-nhe)/N, where *nho* is the number of homogamic matings, *nhe* is the number of heterogamic matings, and *N* the total number of matings. SIIs range from − 1.00 (preference for unlikes, heterogamy) through 0 (random mating) to + 1.00 (preference for likes, homogamy). This experimental design is not devoid of bias. Indeed, when the number of partners is finite and remating is limited, the choice of the last individuals is conditioned by the choice of the previous ones. This could lead to erroneous signals for assortative mating, e.g. when both males and females from the same population tend to mate earlier than those from the other population (Ehrman and Parsons 1981). However, unbiased rates of assortative mating could be estimated by accounting for the order of mating pairs, using a statistical model described extensively in Supporting Methods. This method provides the maximum-likelihood estimates of the assortative mating coefficient (called eSII, or h in the model and Supporting Methods) and accounts for the possibility of remating in males. Several remating rates were tested and did not significantly influence the estimation of SII (see Fig. S3), hence in practice a remating rate of 0.5 was chosen for the general analysis (Tables S2–S4). To avoid convergence issues, the female remating rate was set to 0.001. Two additional parameters (biases in mating order for males and females) were also estimated by the model (Table S5). However, their biological interpretation is not that straightforward as they may correspond to either a general preference for one population of one sex, or a different mating speed between both populations. The statistical departure of the SII from 0 was estimated by a likelihood ratio test. Simulations showed that this procedure efficiently corrects for such mating order biases, and was even slightly more powerful than homogeneity (Fisher) tests when mating order was unbiased.

The model was implemented in R version 3.1.1 (R Core Team 2016), the code is available in Supporting Methods, along with the raw data. A comparison of the uncorrected SII and the model-estimated SII is provided in Supporting Information Fig. S3 and Tables S2–S4. Hereafter, we will refer only to the estimated SII that will be simply called eSII.

### Analysis of cuticular hydrocarbons (CHCs) *via* gas chromatography–ion trap mass spectrometry (GC–MS)

Male virgin flies were collected, isolated from females, and aged to 3 days. Flies were pooled in glass vials and submerged in 1 ml of HPLC-grade hexane (Carl Roth, Germany). Replicates consisted of 10 males per sample. We did nine replicates for A28^wt^ and 10 replicates for each A28^kd^, O11^wt^ and O11^kd^. Two µg of octadecane (C18) per sample was added as the internal standard for absolute quantification of CHCs. Extraction of CHCs was performed for 10 min at room temperature under constant agitation, after which the flies were removed. Extracts were evaporated to about 20–30 µl of hexane under a constant stream of argon, and then transferred to a 150 µl GC-µ-vial (CZT, Germany). 1 µl aliquots were injected into a Varian 450GC gas chromatograph coupled to a Varian 240MS mass spectrometer (Agilent Technologies, Germany). A DB5-MS capillary column (30 m × 0.25 mm diameter, film thickness: 0.25 µm, Agilent Technologies, Germany) was used and the GC was programmed from 150 °C to 300 °C at 15 °C/min with a 27 min final isothermal hold. Helium, with a constant flow rate of 1 ml/min, was used as carrier gas. Recording of mass spectra was performed using electron ionization (EI-MS) in external ionization mode and data acquisition plus quantifications were done with MS Workstation v6.9.3 Software (Agilent Technologies, Germany). Consequently, peaks were identified by their mass spectra in comparison to previously published hydrocarbon profile analyses of *D. paulistorum* (Kim et al. 2004; Chao et al. 2010) and *D. melanogaster* (Ueyama et al. 2005). Hydrocarbon quantities were calculated from peak areas and then centered log-ratio-transformed according to Aitchinson 1986. To test for differences in chemical profiles between wild type and knockdown individuals, principal component analyses (PCAs) based on eigenanalysis of covariances were performed to reduce numbers of variables. Consequently, resulting PCs were used for discriminant analyses (DAs), to test for among-group differences.

### Fluorescence *in situ* hybridization (FISH) on *D. paulistorum* oenocytes

Ten to 15 female and male abdomen per semispecies were dissected in RNase-free 1 × phosphate buffered saline (PBS). After removing inner organs, cuticles were fixed in 3.7% formaldehyde in RNase-free PBS for 20 min at room temperature and consequently washed with PBTX (1 × PBS, 0.3% Triton-X 100). After permeabilization with 70% ethanol overnight at 4 °C, samples were hybridized overnight in 10% formamide, saline sodium citrate (SSC) and 0.5 nmol of CAL Fluor Red 590-labeled customized *Wolbachia 16*-*23S* rRNA probe (Biosearch Technologies, USA; Schneider et al., *in press*). Samples were then washed in 10% formamide and SSC, stained with DAPI-SSC (1 µg/ml) and mounted in Roti®-Mount FluorCare (Carl Roth, Germany) on sterilized microscope slides. Cuticles were analyzed on an Olympus FluoView confocal microscope. Beam paths were adjusted to excitation/emission peaks of used fluorophores: 569/591 nm for CAL Fluor Red 590 (*Wolbachia*), and 350/450 nm for 4′,6-diamidin-2-phenylindol (DAPI). Images were processed with Fiji software (http://fiji.sc). Hybridization experiment was repeated twice and a minimum of 15 flies was assayed in each experiment.

## Results

### Knockdown of obligate *Wolbachia* in *Drosophila paulistorum* semispecies is of transient nature

**Fig. 2:**
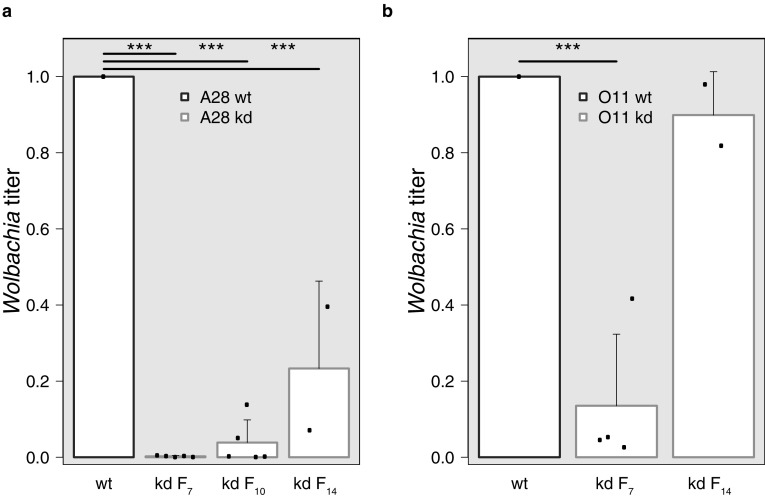
Quantitative analysis of *Wolbachia* titers in wild type (wt) and knockdown (kd) *D. paulistorum* males. Bars represent *Wolbachia* titers measured *via* quantitative real time PCR on DNA from whole body extracts of A28 (**a**), and O11 (**b**) using *Wolbachia 16S* rRNA normalized against *Drosophila* ribosomal protein 17 (*RPS17*). Each purple bar represents the mean *Wolbachia* titer in wt males (mean from 8 to 10 biological replicates is plotted). Orange bars represent titers in F_7_/F_14_ kd males (post treatment); individual biological replicates are shown as black dots. In addition, for A28^kd^, F_10_ titers were tested. Kd-titers are relative to wt ones (set to 1). Each sample was run in technical duplicates. Error bars represent the standard deviation of the biological replicates. Asterisks indicate significance of differences between titer levels. *P* values were calculated using *Fisher’s* exact test. *wt* wild type, *F*_*7*_, *F*_*10*_, *F*_*14*_ fly generations 7, 10 and 14. (Color figure online)

To test whether manipulation of native *Wolbachia* titers and associated disruption of the host-symbiont homeostasis can trigger de novo reproductive isolation (RI) in *D. paulistorum*, we generated *Wolbachia*-knockdown (kd) males of two different semispecies (Amazonian: A28, and Orinocan: O11) by mild rifampicin treatment for three consecutive generations (T1-T3) and in the following generations on regular media without antibiotics (F_1_^kd^–F_14_^kd^) for restoration. For details see Supporting Information Table S1, Fig. S1. Global *Wolbachia* titers of these males (A28^kd^, O11^kd^) were tested against wild type (wt) controls (A28^wt^, O11^wt^) in quantitative real time PCR (qPCR) assays targeting the *Wolbachia 16S* ribosomal RNA gene. Assays obtained from F_7_^kd^ (knockdown generation 7) revealed massive titer reduction to about 5 and 14% of the average native wt *Wolbachia* titers (Fig. 2a, b).

To further assess whether *Wolbachia* titers return to native levels in later generations post kd, we tested symbiont load by F_14_ and earlier generations. Whereas in O11 the global symbiont titer was close to wt-levels at F_14_ (90%; Fig. 2b), A28 flies have gradually reached only 23% of their wt-titer at this time point (Fig. 2a).

### Mate choice assays reveal de novo assortative mating phenotype of wild type (wt) females towards *Wolbachia*-knockdown males

**Fig. 3:**
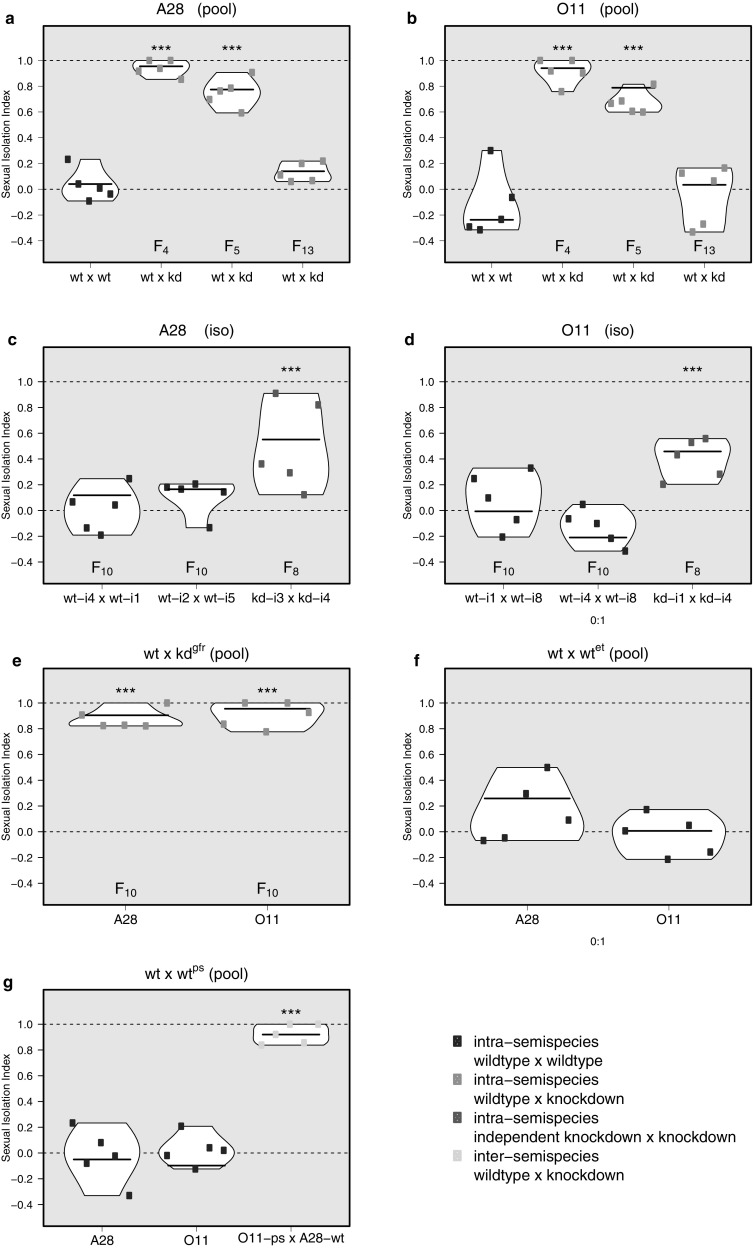
Violin plots showing estimated sexual Isolation Index (eSII) in multiple choice assays between *D. paulistorum* wild type (wt), *Wolbachia*-knockdown (kd), axenic (et), and penicillin/streptomycin-treated (ps) flies. (**a, b**) Intra-semispecific mate choice assays with pool lines. Wild type (wt) flies (control, in purple) or knockdown (kd) flies (in orange) were assayed at generations F_4_, F_5_, F_13_, against wt flies, for A28 and O11. (**c, d**) Intra-semispecific mate choice assays with isofemale lines. wt vs. wt (purple), and kd vs. kd (pink) mating choice assays were performed between different isofemale lines, respectively at generation F_10_ and F_8_. **e** Intra-semispecific assays between wt and gut-flora-restored flies (gfr) performed at generation F_10_ for A28 and O11. **f** Intra-semispecific assays between wt and ethanol-washed wt flies (et) performed at generation F_2_ for A28 and O11. **g** Intra-semispecific assays between wt and penicillin/streptomycin-treated flies (ps), performed at generation F_3_ for the A28 and O11. The last assay (yellow) is an additional inter-semispecific assay between wt and ps flies. For all assays, the model-estimated SII was used on either individual replicates (dots), or considering the 5 replicates (horizontal bars). Asterisks denote assays for which the likelihood ratio test (LRT) is highly significant (*p* values < 10^−4^), the null model being random mating (SII = 0). All the *p* values are reported in Tables S2–S4. (Color figure online)

Among sympatric *D. paulistorum* semispecies, no hybrids are formed inter-semispecifically in nature (Dobzhansky and Spassky 1959). When performing inter-semispecific mate choice assays involving A28^wt^ and O11^wt^ semispecies, we observed strong RI, revealed by a high estimated Sexual Isolation Index (eSII) (Ehrman 1965; Ehrman and Powell 1982; Supporting Information: Table S2, assays 1, 2). In contrast, intra-semispecific control assays showed no assortative mating in standard interbreeding ‘pool’ lines (Fig. 3a, b wt × wt (purple) and Supporting Information Table S2, assays 3, 4). Results of statistical testing are summarized in Table S2.

In order to test our hypothesis that manipulation of the native *Wolbachia* titer and the associated disruption of the host-symbiont homeostasis in *D. paulistorum* might trigger de novo premating isolation intra-semispecifically, we assayed mating behavior of *Wolbachia*-kd pool lines towards wt flies of the same semispecies (Fig. 3a, b, orange, and Table S3, assays 1–6). Assays run with knockdown flies at generation F_4_ or F_5_ post-treatment, consistently showed high eSII, i.e. mating between likes rather than unlikes. This points towards de novo mate discrimination between wt and kd flies from the same semispecies that significantly differ in their symbiont load (kd F_7_ in Fig. 2a, b). However, at generation F_13_ homogamy was less pronounced among all of them, and eSII did not differ significantly from what is expected under random mating. Importantly, although O11 flies showed rapid increase of *Wolbachia* levels by kd F_14_ (Fig. 2b), only a slower, gradual titer increase was observed in A28 (Fig. 2a). Hence the restoration of the random mating phenotype in O11 might directly correlate with the global restoration of the symbiont level at faster pace, whereas the reversion to random mating of A28 flies might depend on a critical titer threshold of *Wolbachia* and/or their tropism to sensitive host tissues such as oenocytes (see discussion).

To rule out that drift effects were responsible for observed de novo sexual isolation, we additionally assayed wt and kd lines that were reared as ten isofemale lines per semispecies (A28^wt-i^, O11^wt-i^ and A28^kd-i^, O11^kd-i^, respectively). First, two out of ten randomly picked control isofemale lines (wt-i) representing each semispecies were tested against each other (intra) in F_10_ in order to verify random mating between them (Fig. 3c, d wt-i × wt-i, purple, and Supporting Information Table S2, assays 5–8). The observed low eSIIs indicated the absence of any drift-dependent sexual isolation. To test for emerging sexual isolation between kd-isofemale lines upon drift (as a response to *Wolbachia*-kd and consequent restoration) we performed intra-semispecific choice assays using independent F_8_ isofemale knockdown lines (kd-i). Surprisingly in this case the assays revealed significant isolation between them (*p* values < 10^−4^; Fig. 3c, d, pink, and Supporting Information Table S3, assays 7, 8).

Taken together, control assays between independent wt isofemale lines rule out drift as a potential factor for triggering de novo premating sexual isolation observed between wt and kd lines, whereas independent knockdown lines have possibly drifted apart from each other upon isolation having potentially different levels and/or individual tissue tropisms of *Wolbachia* during restoration at F_8_. Overall, the emergence of high eSIIs in intrastrain assays suggests that even slight perturbations of the native *Wolbachia* - *D. paulistorum* homeostasis and/or tropism are sufficient to induce de novo RI in this system, even between individual knockdown isofemale lines. This latter and quite intriguing observation, however, will need further investigations, which are beyond the scope of our current analyses (see discussion).

### No evidence for influence of gut microbiota on de novo assortative mating phenotype

Earlier and recent studies, however, have addressed the potential influence of diet and consequently the gut microbiome on host sexual isolation not only in *D. pseudoobscura* (Dodd et al. 1989), but also in highly inbred lines of *D. melanogaster* (Sharon et al. 2010; Ringo et al. 2011). Here, sexual isolation can be triggered by manipulating the fly’s gut microbiome, based on a certain dietary regime. However, two recent studies have questioned the general role for gut bacteria or diet composition in driving reproductive isolation in *D. melanogaster* (Najarro et al. 2015; Leftwich et al. 2017). In the light of these still controversial findings obtained from inbred *D. melanogaster* strains, we have tested *D. paulistorum* gut flora restored (gfr) lines, where kd lines were fed with wt feces, in parallel with our unfed kd lines (see “Methods” for details). Assays with these lines and wt flies revealed almost complete SII suggesting that gut flora restoration does not affect the de novo assortative mating phenotype (*p* values < 10^−4^, Fig. 3e, and Supporting Information Table S4, assays 1, 2). To further exclude an effect of the gut microbiome associated with the egg smear, we assayed axenic flies, i.e., eggs washed with ethanol (et, Fig. 3f, and Supporting Information Table S4, assays 3, 4), and penicillin/streptomycin-treated flies (ps, Fig. 3g, purple, and Supporting Information Table S4, assays 5, 6). Mating behavior with both sets were compatible with random mating (absence of assortative mating). Hence, in contrast to rifampicin, which acts on *Wolbachia* levels, neither sterilizing eggs, nor treatments with penicillin/streptomycin had any effect on mate choice behavior in this system. Furthermore, to test whether penicillin/streptomycin affects mate behavior of females we performed inter-semispecific control assay between wildtype A28 and penicillin/streptomycin-treated O11 flies that still revealed high sexual isolation (ps, Fig. 3g, yellow, Supporting Information Table S4, assay 7, *p* value < 10^−4^), confirming that penicillin/streptomycin-sensitive gut microbes in females have no effect on mating behavior in this system.

Importantly, we are aware that none of our control assays on its own can exclude the spurious presence of some hidden non-*Wolbachia* microbe affecting mate behavior. However, the combination of three independent control assays, i.e., gut-flora restoration, egg-surface sterilization and penicillin/streptomycin-treatment, strongly supports our hypothesis that endosymbiotic *Wolbachia*, which are resilient to penicillin/streptomycin and egg surface sterilizing treatments (Audsley et al. 2017; Leclerq et al. 2017; Ye et al. 2017), are interfering with mate discrimination of *D. paulistorum* in both sexes and not any other bacteria.

Concordantly, only rifampicin treatment massively reduces *Wolbachia* load and induces de novo assortative mating behavior of wildtype females against knockdown males from the same semispecies (this study) that also selectively triggers loss of assortative mating behavior in knockdown females against males from a different semispecies (Fig. 3g, yellow; Miller et al. 2010).

### Sexual pheromone profiles are altered in *D. paulistorum* knockdown males

Based on the observed de novo assortative mating phenotype, we tested whether the pheromone composition of *Wolbachia*-kd males was altered in comparison with wt males. Males from all six *D. paulistorum* semispecies exhibit characteristic pheromone profiles (all share the same 15 major compounds but in varying quantities), including four male-specific long-chain esters, which are recognized by females as a semispecific blend because of the differences in their relative concentrations (Supporting Information Fig. S4; Kim et al. 2004; Chao et al. 2010). In contrast to other *Drosophila* species such as *D. melanogaster*, no female-specific compounds are known in *D. paulistorum* (Chao et al. 2010) and mate decisions appear—to our current knowledge—to be exclusively made by females (Ehrman 1965; Ehrman and Parsons 1981). Here we extended hydrocarbon profile analyses to a total of 27 compounds found in all tested semispecies by adding two new, unknown compounds (Supporting Information Table S6, compounds 9, 13) and by resolving the original C33, C35, and C37 peak clusters (C = chain length of the compound; 33, 35, and 37 carbon atoms).

**Fig. 4:**
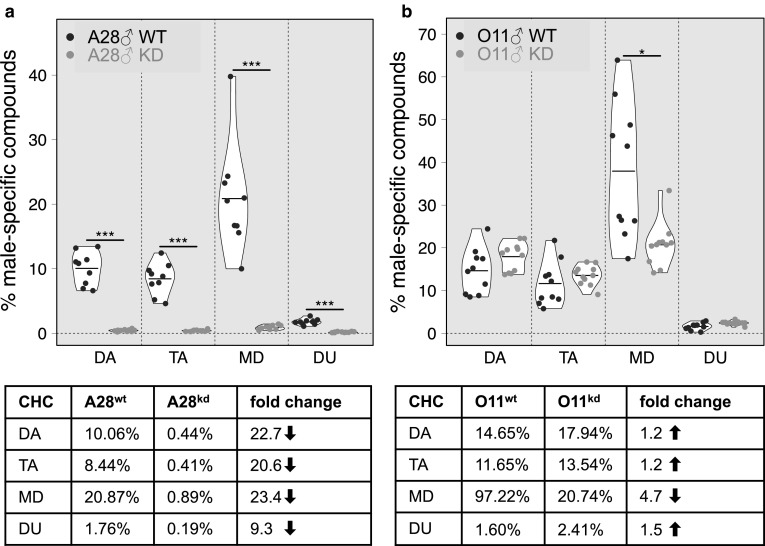
Quantitative changes in *D. paulistorum* male-specific pheromone compounds upon *Wolbachia*-knockdown. Violin plots show percentage of the four male-specific ester compounds of the total pheromone profiles (27 compounds) of A28 (**a**) and O11 (**b**). Individual replicates for wild type controls (wt) are shown in purple, and in orange for F_7_ knockdown males (kd). Horizontal bars correspond to the mean. Tables below violin plots list corresponding percent change of compounds plus n-fold increase (↑) or decrease (↓) between wt and kd. Asterisks indicate statistical significance based on two-tailed *p* values from *Student’s t* tests. *DA* 11-docosenyl-acetate, *TA* 19-triacontenyl-acetate, *MD* methyl-(Z)-tetradecanoate, *DU* di-unsaturated acetate C_32_H_60_O_2_. (Color figure online)

In full agreement with earlier reports (Kim et al. 2004; Chao et al. 2010), discriminant analyses of sex pheromone profiles from A28^wt^ and O11^wt^ males revealed major differences among the semispecies (Supporting Information Fig. S5). In order to test for differences in male pheromone profiles upon *Wolbachia*-kd, we compared profiles between F_7_ kd males and wt males for each semispecies. Based on the 27 quantified components (Supporting Information Table S6), six principal components were extracted capturing 84.9% of the total variance. The discriminant analysis revealed statistically significant differences between both semi-species A28^wt^ and O11^wt^ males (Wilks’ Lambda = 0.178, X^2^ = 24.2, df = 6, *p* < 0.001) as well as between A28^wt^ and A28^kd^ males (Wilks’ Lambda = 0.059, X^2 ^= 39.6, df = 6, *p* < 0.001) and also between O11^wt^ and O11^kd^ (Wilks’ 446 Lambda = 0.117, X^2 ^= 34.3, df = 6, *p* < 0.001, Supporting Information Fig. S5).To further analyze differences in pheromone profiles between wt and kd males, we calculated the relative quantities of the four male-specific compounds in *D. paulistorum* male profiles (11-docosenyl-acetate, DA, 19-triacontenyl-acetate, TA, di-unsaturated acetate C_32_H_60_O_2_, DU, and methyl-(Z)-tetradecanoate, MD). As shown in Fig. 4, we observed most drastic changes in quantities of these compounds in A28^kd^ males, where all four male-specific compounds were decreased between 9- and 23-fold when compared to wt levels (Fig. 4a, *p* < 0.0001 for all four compounds). Although not as drastic as A28^kd^, in O11^kd^ males at least one male-specific compound also showed significant changes: compared to O11^wt^ males, MD was reduced fivefold (Fig. 4b, *p* = 0.0399).

### *Wolbachia* colonize oenocytes in *D. paulistorum*

**Fig. 5:**
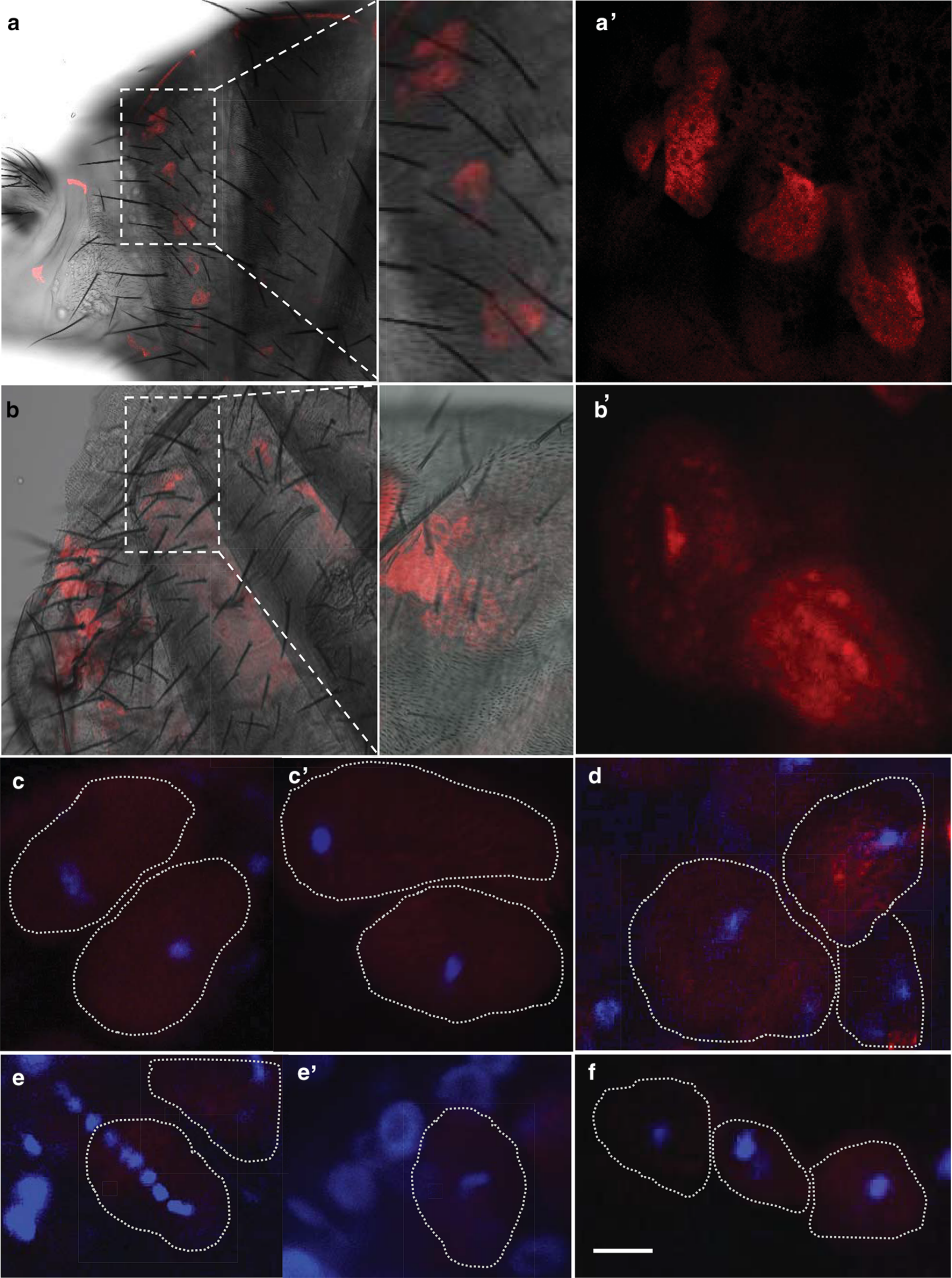
Fluorescence *in situ* hybridization (FISH) on *Drosophila* oenocytes. (**a, a**′) *Wolbachia* are present in *D. paulistorum* O11^wt^ females, and (**b, b**′) in O11^wt^ males. **c** Symbionts are lacking from oenocytes of O11 F_4_^kd^ males and from F_4_^kd^ females (**c**′). **d** In F_13_ post kd, oenocytes seem recolonized by *Wolbachia*. (**e, e**′) Oenocytes from *Wolbachia*-infected *D. melanogaster* Harwich strain (H2) tested also negative. **f***Wolbachia*-uninfected *D. willistoni* (Willi3) was used as negative control. *Wolbachia* are shown in red (*16-23S* rRNA-specific probe); *Drosophila* nuclei are stained in blue (DAPI). Grey dashed lines mark borders of oenocyte cells. *Wol Wolbachia, kd* knockdown. Scale bar is 10 µm. (Color figure online)

In insects, pheromone components are mainly synthesized in specialized cells located under the fly’s cuticle, the oenocytes (reviewed in Blomquist and Bagnères 2010; Chung and Carroll 2015; Dembeck et al. 2015). We hypothesized that these specialized cells could serve a primary somatic target for *Wolbachia* to manipulate host pheromone profiles in *D. paulistorum*. Since male pheromone profiles were altered upon *Wolbachia* kd, we tested the infection status of male oenocytes in both *D. paulistorum* wt and kd individuals. As shown in Fig. 5, oenocytes from O11^wt^ females (Fig. 5a, a′) and males (Fig. 5b, b′) harbor *Wolbachia*. We could also detect the symbiont in oenocytes of A28^wt^ males (not shown). Post *Wolbachia*-kd oenocytes, however, are cleared from the symbiont (F_4_ O11^kd^; males in Fig. 5c; females in c′). Since our mate choice assays indicated reversion to wt mating behavior, i.e., loss of de novo assortative mating, around F_13__/__14_ post *Wolbachia*-kd, we tested whether this phenotype correlates with a potential recolonization of *Wolbachia* to oenocytes around this generation post kd. As shown in Fig. 5d, oenocytes of F_13_ O11^kd^ males are, at least partially, recolonized by the endosymbiont. Oenocytes of other *Drosophila* hosts like the *w*Mel-infected *D. melanogaster* strain Harwich-2, (males in Fig. 5e, females in e′) in which *Wolbachia* have evolved facultative symbiotic interactions and do not affect mate behavior, are devoid of the symbiont. This is in contrast to *D. paulistorum*, where *w*Pau *Wolbachia* are obligate mutualists affecting host behavior (Miller et al. 2010 and this study). Oenocytes derived from the *Wolbachia*-uninfected *D. willistoni* strain Willi3, a sister species of *D. paulistorum*, were used as negative controls (Fig. 5f). These data suggest that the oenocyte-tropism of *w*Pau is a phenotypic specificity for the *Wolbachia-D. paulistorum* system.

## Discussion

### *Wolbachia*-knockdown provokes a de novo assortative mating phenotype

*D. paulistorum* semispecies are very sensitive to standard antibiotic treatments (Ehrman 1968; Kernaghan and Ehrman 1970) since in this symbiosis, *Wolbachia* is a fixed obligate mutualist providing vital, but still undetermined functions for its native hosts (Miller et al. 2010). Similar *Wolbachia*-dependencies were found in the parasitoid wasp *Asobara tabida* (Dedeine et al. 2001; Pannebakker et al. 2007), bedbugs of the genus *Cimex* (Hosokawa et al. 2010; Nikoh et al. 2014; Moriyama et al. 2015), and in the rice water weevil *Lissorhoptrus oryzophilus* (Chen et al. 2012). Furthermore, even partial depletion of fixed mutualistic *Wolbachia* from filarial nematodes affects host fitness and fecundity but also triggers overexpression of host nuclear and mitochondrial genes in order to partially compensate the loss of some gene functions provided by *Wolbachi*a (Pfarr et al. 2008; Strübing et al. 2010).

Here we have assayed for behavioral consequences of symbiont knockdowns in males of two different *D. paulistorum* semispecies, i.e., *Wolbachia* low-titer Amazonian (A28) and high titer Orinocan (O11) strains. Our data strongly suggest that in both systems partial knockdown of the endosymbiont in males significantly affects their attractiveness for homogamic wt-females, which seems to correlate with global titer levels. However, restoration to random mating in later generations post treatment (Fig. 3a, b) seems to correlate with the gradual quantitative reconstitution of global symbiont titers that are fast in O11 (Fig. 2b), but not in A28 (Fig. 2a). In our assays, A28 flies expressing random mating at F_13_ still show significantly lower global *Wolbachia* levels than wt flies. We speculate that reversion to random mating of A28 flies observed at later generations post-treatment might not depend on the global restoration of the symbiont titer in this semispecies to wt, but possibly because of a critical titer threshold and/or the symbiont’s tropism to sensitive tissues and organs associated with mating behavior. In order to test this hypothesis, however, detailed temporal and spatial *in situ* quantifications of the symbiont in different host tissues during the restoration phase are required, which were beyond the scope of our current study.

As shown by this and earlier studies, under laboratory conditions different *D. paulistorum* semispecies express strong assortative mating behavior against each other, which is sensitive to rifampicin and tetracycline (Miller et al. 2010), but insensitive to penicillin and streptomycin that do not target the *Wolbachia* endosymbiont (this study). It seems feasible although not tested that strict female mate choice might prevent mating between incompatible members of different *D. paulistorum* semispecies also in nature. If true, this behavior has most likely coevolved in conjunction with the obligate endosymbiont to avoid detrimental reproductive phenotypes like hybrid mortality and male sterility in crosses between inter-semispecies (Ehrman 1968; Kim and Ehrman 1998). Here we have evaluated the potential of *Wolbachia* to trigger assortative mating behavior in *D. paulistorum* towards *per se* compatible mates under laboratory conditions. Our results suggest that kd-males appear sexually less attractive for their wt-female counterparts in mate choice assays upon manipulation of the native *w*Pau-*Wolbachia* titer since they were expressing high SIIs in all assays. Such high SIIs were previously found only between members of different *D. paulistorum* semispecies (Ehrman 1965; Malogolowkin-Cohen et al. 1965) or between *Drosophila* species that are sexually isolated from each other (reviewed in Spieth and Ringo 1983; Martin and Hosken 2003; Anderson and Kim 2005; Castrezana and Markow 2008). Our data also indicate that de novo sexual isolation in *D. paulistorum* under our experimental conditions is transient as eSIIs reverted to random mating around generation F_13_ post kd (only 45–57% homogamy) together with the observed re-colonization of male oenocytes. However, experimental reduction of native *D. paulistorum*-*Wolbachia* titers was sufficient to alter males so that they were subsequently rejected by wt-females in the two semispecies tested, at least transiently. Interestingly we observed significant assortative mating between independently established kd-isofemale lines of the same semispecies, a finding most likely caused by drift. In such, heterogeneity of kd isolines could be a result of different temporal and spatial dynamics of *Wolbachia* recolonization of behavioral important tissues in the individual lines post knockdown. To test this drift hypothesis for kd lines, however, detailed quantitative and qualitative analyses of global symbiont titer levels, but also their *in situ* tropism and densities will be necessary from multiple staged flies and tissues of the same generation. In our current study, extensive analyses like these were not possible due to limitations of fly material at this time point.

### Host pheromonal signatures may be a target for *Wolbachia* to signal the infection state and to impact mate choice

In many insects, sexual pheromones serve as recognition cues for mate choice between and within species and are hence important players in reproductive isolation (Coyne et al. 1994; Savarit et al. 1998; Ferveur 2005). Moreover, such cues might even be prone to manipulation by pathogenic bacteria, as recently shown in *D. melanogaster* (Keesey et al. 2017).

In our study, the observed alteration of pheromone blends in kd-males may explain why females prefer wt-mates carrying the intact native profile. This finding is corroborated by studies showing that in lekking sandflies (*Lutzomya*), female mate choice is influenced by the amount of pheromones released by males before potential mating (Jones and Hamilton 1998; Jones et al. 2000). Another study demonstrated the influence of *Wolbachia* on host odor-linked mate preference in the terrestrial isopod *Armadillidium* vulgare (Richard 2017). In A28^kd^ and O11^kd^ males, relative quantities of pheromone compounds were affected differently, suggesting that the quantitative change in at least one of the male-specific components, but possibly also in conjunction with some other non-sex-specific CHCs, might be sufficient to trigger rejection of kd-males by wt-females in choice experiments. Importantly, our findings are in line with a recent study suggesting that *Wolbachia* can influence pupal communication between females and males in *Drosophila melanogaster* by modulating CI levels (Pontier and Schweissguth 2015, 2017; but also see; Jacquet et al. 2017). However, as shown earlier, facultative *Wolbachia* symbionts of *D. melanogaster* adults do not affect mate choice behavior in this system at all, but possibly other gut microbes (Sharon et al. 2010; Ringo et al. 2011; Arbuthnott et al. 2016). Importantly, a very recent study has severely questioned the potential impact of the gut microbiome on reproductive isolation since the microbiome of *D. melanogaster* is not fixed but reported as flexible and environmental determined (Leftwich et al. 2017). In the mutualistic *D. paulistorum-Wolbachia* system, however, where the endosymbiont is fixed by serving vital but still undetermined functions to their native host (Miller et al. 2010) we suggest that these obligate *Wolbachia* have also a direct or indirect impact on adult cuticular hydrocarbon (CHC) profiles, where male-specific and sex-unspecific compounds change quantitatively upon *Wolbachia*-kd in adult males.

The presence of *Wolbachia* in oenocytes of O11^wt^ males implies a direct or indirect interaction of the symbiont with host pheromone production and mate choice behavior as a consequence. The potential effects of the endosymbiont in oenocytes on female pheromone blending, however, await elucidation. If the presence of *Wolbachia* in male oenocytes is essential to express the semispecies-specific pheromone profile, loss of the symbiont from male oenocytes (F_4_ post kd) might explain why pheromone profiles are altered and knockdown males are consequently rejected as mate partners from homogamic wt-females. Based on our mate choice assays, the de novo assortative mating phenotype behavior is transient and results in reversion to a wt-like situation around F_13_/F_14_ post knockdown. Assuming a direct link between *Wolbachia* tropism in the oenocytes and expression of the male pheromone profile, we expected a recolonization of oenocytes in parallel with reversion to random mating phenotype. We could confirm such recolonization, and thus potentially explain reversion to random mating, in oenocytes of F_13_ O11^kd^ males. Our finding is particularly interesting in the light of the contrasting situation in *D. melanogaster*, where *Wolbachia* do not play a role in mate choice (Sharon et al. 2010; Ringo et al. 2011; Arbuthnott et al. 2016). In our experimental setup, we did not detect *Wolbachia* in *D. melanogaster* oenocytes, which most likely explains why the symbiont does not affect mate choice and pheromone expression in this model system (Sharon et al. 2010). Finally, in contrast with the strict neurotropism of obligate *Wolbachia* to defined brain regions of *D. paulistorum*, (Strunov et al. 2017) native *Wolbachia* are randomly dispersed at low densities in *D. melanogaster* brains (Albertson et al. 2009). However, it remains to be elucidated how *Wolbachia* manipulate male pheromone expression and female mate choice in *D. paulistorum*.

To conclude, our combined data strongly imply that artificial reduction of the obligate *Wolbachia* endosymbiont of *D. paulistorum* males significantly reduces mating success with homogamic wildtype females belonging to the same semispecies. Although these and earlier findings in *D. paulistorum* lead to interpretations based on associations rather than causation and their functional and molecular bases are still undetermined, we propose the following model for the potential impact of the *D. paulistorum* endosymbiont on RI and host speciation in this system. In contrast to most facultative *Wolbachia*-insect associations, where the symbiont is not fixed and does not serve vital host functions, *D. paulistorum* semispecies have evolved vital mutualistic associations with their endosymbiont (Miller et al. 2010) as well as strict tissue tropisms to reproductive host organs, such as the primordial germline cells of embryos and adult gonads (Miller et al. 2010), but also to defined larval and adult brain regions associated with sexual behavior (Strunov et al. 2017) and pheromone producing oenocytes (this study). As implicated from the results of this study even partial depletions of the mutualist from their primary somatic host targets, such as oenocytes, might directly or indirectly alter pheromone signatures of males, which are no longer accepted as mates from stress-free wt females.

It appears likely that spontaneous symbiont knockdowns might also happen in the wild by stochastic exposure of the host to natural antibiotics or heat stress. This de novo assortative phenotype expressed by females against aberrant homogamic males can significantly disrupt gene flow within populations via premating isolation, at least transiently. As shown earlier, however, *Wolbachia*-kd males are accepted by kd females, which randomly mate even with heterogamic males under lab conditions (Miller et al. 2010). Under this scenario two sexually isolated reproductive groups would emerge and coexist next to each other. Since population sizes of *Wolbachia* have dropped significantly upon such external stresses, it seems quite likely that genetic drift (Chrostek et al. 2013; Schneider et al. 2013; Newton et al. 2014) can cause disruptive diversification of earlier-compatible *Wolbachia* variants that consequently trigger high cytoplasmic incompatibilities plus complete male sterility at postmating levels.

Hence it seems plausible that in Dobzhansky´s earlier studies (Dobzhansky and Pavlovsky 1966, 1971, 1975) where spontaneous emergence of strong post mating incompatibilities between long term isolated sub lines of the same *D. paulistorum* semispecies were observed, he had actually detected the final outcome of this symbiotic stress and drift effect, observed in our current experimental study. Planned studies, however, should elucidate the mechanistic basis of *Wolbachia*-influence on male pheromone production and whether similar scenarios observed under lab conditions also take place in nature.

## Electronic supplementary material

Below is the link to the electronic supplementary material.

Supplementary material 1 (EPS 1177 KB)

Supplementary material 2 (EPS 4793 KB)

Supplementary material 3 (EPS 1796 KB)

Supplementary material 4 (EPS 4078 KB)

Supplementary material 5 (PPTX 46 KB)

Supplementary material 6 (DOCX 96 KB)

Supplementary material 7 (DOCX 115 KB)

Supplementary material 8 (DOCX 104 KB)

Supplementary material 9 (DOCX 100 KB)

Supplementary material 10 (DOCX 168 KB)

Supplementary material 11 (DOCX 105 KB)

Supplementary material 12 (PDF 243 KB)
