# Supplementary material for: Symbiont-Driven Male Mating Success in the Neotropical Drosophila paulistorum Superspecies
Source: Behav Genet. 2018 Nov 19;49(1):83–98. doi: 10.1007/s10519-018-9937-8 (PMC6327003; doi:10.1007/s10519-018-9937-8)
Supplement: Supplementary file 5 — Supplementary material 5 (PPTX 46 KB) [file 10519_2018_9937_MOESM5_ESM.pptx]

## Slide 1
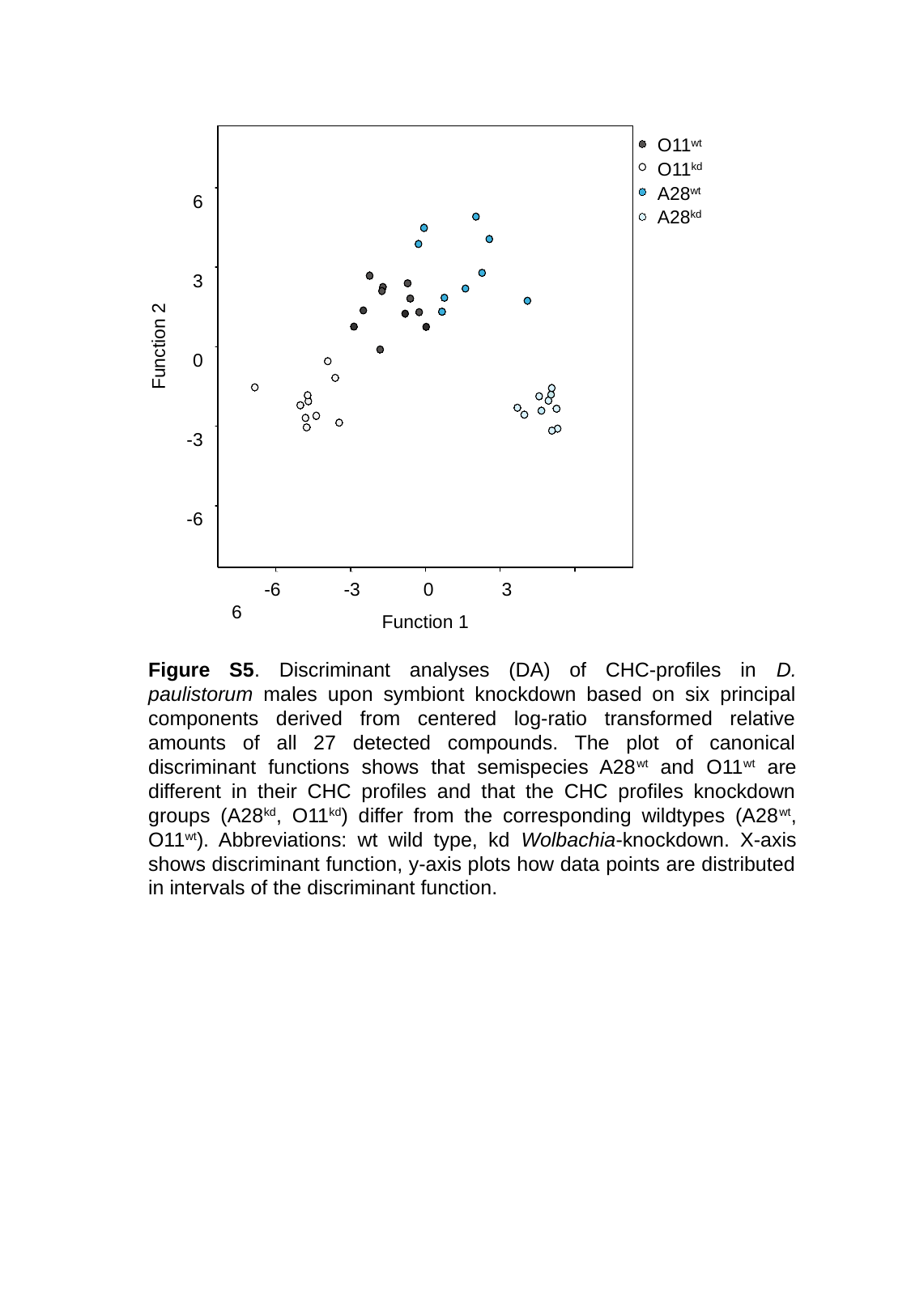

6
3
0
-
3
-
6
-
6
-
3
0
3
6
Function 2
O11wt
O11kd
A28wt
A28kd
6
3
0
-3
-6
 -6 -3 0 3 6
Function 1
Figure S5. Discriminant analyses (DA) of CHC-profiles in D. paulistorum males upon symbiont knockdown based on six principal components derived from centered log-ratio transformed relative amounts of all 27 detected compounds. The plot of canonical discriminant functions shows that semispecies A28wt and O11wt are different in their CHC profiles and that the CHC profiles knockdown groups (A28kd, O11kd) differ from the corresponding wildtypes (A28wt, O11wt). Abbreviations: wt wild type, kd Wolbachia-knockdown. X-axis shows discriminant function, y-axis plots how data points are distributed in intervals of the discriminant function.
