# Supplementary material for: Symbiont-Driven Male Mating Success in the Neotropical Drosophila paulistorum Superspecies
Source: Behav Genet. 2018 Nov 19;49(1):83–98. doi: 10.1007/s10519-018-9937-8 (PMC6327003; doi:10.1007/s10519-018-9937-8)
Supplement: Supplementary file 6 — Supplementary material 6 (DOCX 96 KB) [file 10519_2018_9937_MOESM6_ESM.docx]

| Abbreviation | Line name | Semispecies | Description |
| --- | --- | --- | --- |
| A28^wt^ | control line A28 | Amazonian | wild type |
| O11^wt^ | control line O11 | Orinocan | wild type |
| A28^wt-i^ | control line A28 | Amazonian | wild type, isofemale line |
| O11^wt-i^ | control line O11 | Orinocan | wild type, isofemale line |
| A28^kd^ | knockdown line A28 | Amazonian | 0.2% rifampicin for 3 gen plus restoration for 3 gen, pool line |
| O11^kd^ | knockdown line O11 | Orinocan | 0.2% rifampicin for 3 gen plus restoration for 3 gen, pool line |
| A28^kd-i^ | knockdown line A28 | Amazonian | 0.2% rifampicin for 3 gen plus restoration for 3 gen, isofemale line |
| O11^kd-i^ | knockdown line O11 | Orinocan | 0.2% rifampicin for 3 gen plus restoration for 3 gen, isofemale line |
| A28^gfr^ | gut flora restored line A28 | Amazonian | 0.2% rifampicin for 3 gen plus restoration for 3 gen, gut flora restoration, pool line |
| O11^gfr^ | gut flora restored line O11 | Orinocan | 0.2% rifampicin for 3 gen plus restoration for 3 gen, gut flora restoration, pool line |
| A28^et^ | axenic line A28 | Amazonian | eggs washed with 70% ethanol before establishment of line |
| O11^et^ | axenic line O11 | Orinocan | eggs washed with 70% ethanol before establishment of line |
| A28^ps^ | pen/strep line A28 | Amazonian | 0.01% penicillin/streptomycin for 1 gen |
| O11^ps^ | pen/strep line O11 | Orinocan | 0.01% penicillin/streptomycin for 1 gen |

**Table S1. Detailed information on experimental *Drosophila paulistorum* lines.** Pool lines were maintained *via* sibling mating. Rifampicin and penicillin/streptomycin concentration is w/v final concentration in fly food. Abbreviations: gen generation(s), wt wildtype, kd *Wolbachia* knockdown, wt-i wildtype isofemale line, kd-i *Wolbachia* knockdown isofemale line, gfr gut flora restored, et axenic (ethanol-washed) flies, ps penicillin/streptomycin-treated flies.
