# Supplementary material for: Symbiont-Driven Male Mating Success in the Neotropical Drosophila paulistorum Superspecies
Source: Behav Genet. 2018 Nov 19;49(1):83–98. doi: 10.1007/s10519-018-9937-8 (PMC6327003; doi:10.1007/s10519-018-9937-8)
Supplement: Supplementary file 7 — Supplementary material 7 (DOCX 115 KB) [file 10519_2018_9937_MOESM7_ESM.docx]

| Assay | | Semispecies | Strain | Year | eSII (CI 95%) | LRT *p* value | uSII ± SE | *Fisher’s test p value* |
| --- | --- | --- | --- | --- | --- | --- | --- | --- |
| Wildtype control assays (pool) | | | | | | | | |
| 1* | inter | Amazonian/Orinocan | A28^wt^ x O11^wt^ | 1965 | NA | NA | +0.61 ± 0.07 | NA |
| 2 |  | Amazonian/Orinocan | A28^wt^ x O11^wt^ | this study | +0.71 (0.58 – 0.82) | 0 | +0.70 ± 0.07 | < 10^-4^ |
| 3 | intra | Amazonian/Amazonian | A28^wt^ x A28^wt^ | this study | +0.03 (-0.15 - 0.21) | 0.7337 | +0.03 ± 0.09 | 0.8552 |
| 4 |  | Orinocan/Orinocan | O11^wt^ x O11^wt^ | this study | -0.16 (-0.33 – 0.02 | 0.0796 | -0.12 ± 0.09 | 0.2716 |
| Wildtype control assays (isofemale) | | | | | | | | |
| 5 | intra | Amazonian/Amazonian | A28^wt-i4^x A28^wt-i1^ | this study | +0.02 (-0.14 – 0.18) | 0.7661 | -0.03 ± 0.09 | 0.8523 |
| 6 |  | Amazonian/Amazonian | A28^wt-i2^x A28^wt-i5^ | this study | +0.11 (-0.07 – 0.29) | 0.2195 | +0.07 ± 0.09 | 0.5839 |
| 7 |  | Orinocan/Orinocan | O11^wt-i1^ x O11^wt-i8^ | this study | +0.07 (-0.1 – 0.25) | 0.4239 | +0.13 ± 0.09 | 0.2001 |
| 8 |  | Orinocan/Orinocan | O11^wt-i4^ x O11^wt-i8^ | this study | -0.15 (-0.32 – 0.03) | 0.1102 | -0.10 ± 0.09 | 0.3590 |

**Table S2.** **Control mate choice assays between (inter) and within (intra) wild type *D. paulistorum* semispecies.** Estimated and uncorrected Sexual Isolation Index (eSII and uSII) in intra or interspecific control assays. The eSII has been estimated for a male remating rate of 0.5. Other estimated parameters are shown in Table S5. The uSIIs (and associated Fisher’s tests) are indicated for comparison. The assay marked with an asterisk (1) was performed by L. Ehrman in a previous study (Kim et al. 2004). Inter-specific assays (Amazonian (A28) *vs.* Orinocan (O11; assays 1,2)) show high SIIs. Assays 3-4 correspond to mate choice assays performed between members of the same semispecies (intra-semispecific assays). As expected, no isolation is observed when testing A28 *vs*. A28 (assay 3) and O11 *vs*. O11 (assay 4). Assays 5-8 are intra-semispecific assays between independent isofemale lines of A28 (assays 5, 6) and O11 (assays 7, 8), which show no sexual isolation. Abbreviations: wt wildtype, wt-i wildtype isofemale line, CI confidence interval, LRT Likelihood Ratio Test, ±SE standard error of the mean. Fisher’s tests are two-tailed tests. Raw data are available in the supplementary file Raw_Data.
