# Supplementary material for: Symbiont-Driven Male Mating Success in the Neotropical Drosophila paulistorum Superspecies
Source: Behav Genet. 2018 Nov 19;49(1):83–98. doi: 10.1007/s10519-018-9937-8 (PMC6327003; doi:10.1007/s10519-018-9937-8)
Supplement: Supplementary file 8 — Supplementary material 8 (DOCX 104 KB) [file 10519_2018_9937_MOESM8_ESM.docx]

| Assay | | Semispecies | Strain | Generation post treatment | eSII (CI 95%) | LRT *p* value | uSII ± SE | *Fisher’s test p value* |
| --- | --- | --- | --- | --- | --- | --- | --- | --- |
| knockdown assays (pool) | | | | | | | | |
| 1 | intra | Amazonian/Amazonian | A28^kd^x A28^wt^ | 4 | +0.94 (0.87 - 0.98) | < 10^-4^ | +0.93 ± 0.03 | < 10^-4^ |
| 2 |  | Amazonian/Amazonian | A28^kd^x A28^wt^ | 5 | +0.75 (0.62 - 0.86) | < 10^-4^ | +0.77 ± 0.06 | < 10^-4^ |
| 3 |  | Amazonian/Amazonian | A28^kd^x A28^wt^ | 13 | +0.13 (-0.05 - 0.31) | 0.1473 | +0.13 ± 0.09 | 0.2008 |
| 4 |  | Orinocan/Orinocan | O11^kd^ x O11^wt^ | 4 | +0.92 (0.83 - 0.97) | < 10^-4^ | +0.92 ± 0.04 | < 10^-4^ |
| 5 |  | Orinocan/Orinocan | O11^kd^x O11^wt^ | 5 | +0.71 (0.57 - 0.82) | < 10^-4^ | +0.70 ± 0.07 | < 10^-4^ |
| 6 |  | Orinocan/Orinocan | O11^kd^x O11^wt^ | 13 | -0.05 (-0.21 - 0.12) | 0.6008 | -0.10 ± 0.09 | 0.3571 |
| knockdown assays (isofemale) | | | | | | | | |
| 7 | intra | Amazonian/Amazonian | A28^kd-i3^ x A28^kd-i4^ | 8 | +0.51 (0.34 - 0.65) | < 10^-4^ | +0.50 ± 0.08 | 0.0000 |
| 8 |  | Orinocan/Orinocan | O11^kd-i1^ x O11^kd-i4^ | 8 | +0.46 (0.28 - 0.61) | < 10^-4^ | +0.48 ± 0.08 | 0.0000 |

**Table S3. Induction of *de novo* assortative mating in *D. paulistorum* semispecies upon *Wolbachia* knockdown.** Estimated and uncorrected Sexual Isolation Index (eSII and uSII) in intraspecific assays between wildtype and knockdown strains or between different isofemale knockdown strains. The eSII has been estimated for a male remating rate of 0.5. Other estimated parameters are shown in Table S6. Assays were performed with pool lines (1-6) and isofemale lines (7-8) from A28 and O11. Intra-semispecific pool assays in generations F_4_ and F_5_ show high SIIs, whereas in F_13_, SIIs are significantly lower. Intra-semispecific assays between kd-i lines show moderate levels of sexual isolation. Abbreviations: wt wildtype, kd *Wolbachia* knockdown, kd-i *Wolbachia* knockdown isofemale line, CI confidence interval, LRT Likelihood Ratio Test, ±SE standard error of the mean. Fisher’s tests are two-tailed tests (on SII). Raw data are available in the supplementary file Raw_Data.
