# Supplementary material for: Symbiont-Driven Male Mating Success in the Neotropical Drosophila paulistorum Superspecies
Source: Behav Genet. 2018 Nov 19;49(1):83–98. doi: 10.1007/s10519-018-9937-8 (PMC6327003; doi:10.1007/s10519-018-9937-8)
Supplement: Supplementary file 9 — Supplementary material 9 (DOCX 100 KB) [file 10519_2018_9937_MOESM9_ESM.docx]

| Assay | | Semispecies | Strain | Generation  post  treatment | eSII (CI 95%) | LRT  *p* value | uSII ± SE | Fisher’s test  *p* value |
| --- | --- | --- | --- | --- | --- | --- | --- | --- |
| Gut flora-restored assays (gfr) | | |  |  |  |  |  |  |
| 1 | intra | Amazonian/Amazonian | A28^gfr^ x A28^wt^ | 10 | 0.88 (0.78 – 0.95) | < 10^-4^ | +0.88 ± 0.040 | < 10^-4^ |
| 2 |  | Orinocan/Orinocan | O11^gfr^ x O11^wt^ | 10 | 0.92 (0.83 – 0.97) | < 10^-4^ | +0.92 ± 0.040 | < 10^-4^ |
| Axenic (ethanol-washed) assays (et) | | |  |  |  |  |  |  |
| 3 | intra | Amazonian/Amazonian | A28^et^ x A28^wt^ | 2 | 0.18 (0 – 0.35) | 0.0523 | +0.13 ± 0.090 | 0.2008 |
| 4 |  | Orinocan/Orinocan | O11^et^ x O11^wt^ | 2 | -0.02 (-0.18 – 0.15) | 0.8262 | -0.03 ± 0.090 | 0.8564 |
| Penicillin/streptomycin-treated assays (ps) | | |  |  |  |  |  |  |
| 5 | intra | Amazonian/Amazonian | A28^ps^ x A28^wt^ | 1 | -0.03 (-0.21 – 0.15) | 0.7468 | -0.02 ± 0.090 | 1.0000 |
| 6 |  | Orinocan/Orinocan | O11^ps^ x O11^wt^ | 1 | 0.02 (-0.15 – 0.18) | 0.8272 | +0.10 ± 0.090 | 0.3546 |
| 7 | inter | Amazonian/ Orinocan | A28^wt^ x O11^ps^ | 1 | 0.92 (0.83 – 0.98) | < 10^-4^ | +0.93 ± 0.030 | < 10^-4^ |

**Table S4. Mate choice assays with gut flora restored (gfr), axenic (et), and penicillin/streptomycin-treated (ps) *D. paulistorum* semispecies.** Estimated and uncorrected Sexual Isolation Index (eSII and uSII) in assays between wildtype and treated lines. The eSII has been estimated for a male remating rate of 0.5. Other estimated parameters are shown in Table S5. Intra-semispecific assays with gfr lines (assays 1-1: gfr x wt) show high SIIs (similar to assays with knockdown lines). Gfr lines came from direct treatment with faeces of naturally infected flies to consequently restore the gut flora. Intrasemispecific

assays between F1 axenic (et) and wildtype (wt) flies revealed absence of assortative mating (assays 3-4). Intra-semispecific assays with

penicillins/streptomycin-treated (ps) flies showed the same trend (assays 5-6). Assay 7 is an inter-semispecific control assay between A28wtx O11ps, showing

a high SIIs. Abbreviations: wt wildtype, gfr gut flora restored, et axenic, ps penicillin/streptomycin-treated, CI confidence interval, LRT Likelihood Ratio Test,

SE standard error of the mean. Fisher’s exact tests are two-tailed tests. Raw data are available in the supplementary file Raw_Data.
