# Supplementary material for: Symbiont-Driven Male Mating Success in the Neotropical Drosophila paulistorum Superspecies
Source: Behav Genet. 2018 Nov 19;49(1):83–98. doi: 10.1007/s10519-018-9937-8 (PMC6327003; doi:10.1007/s10519-018-9937-8)
Supplement: Supplementary file 10 — Supplementary material 10 (DOCX 168 KB) [file 10519_2018_9937_MOESM10_ESM.docx]

|  | Cross | Table | Assay | eSII | *p* value (eSII) | pM | *p* value (pM) | pF | *p* value (pF) |
| --- | --- | --- | --- | --- | --- | --- | --- | --- | --- |
| 1 | AxO_control | S2 | 2 | 0.71 (0.58 - 0.82) | 0.0000 | 0.35 (0.17 - 0.57) | 0.1773 | 0.65 (0.54 - 0.74) | 0.0055 |
| 2 | AxA_control | S2 | 3 | 0.03 (-0.15 - 0.21) | 0.7337 | 0.46 (0.36 - 0.55) | 0.3750 | 0.57 (0.47 - 0.67) | 0.1660 |
| 3 | OxO_control | S2 | 4 | -0.16 (-0.33 - 0.02) | 0.0796 | 0.60 (0.50 - 0.70) | 0.0590 | 0.58 (0.47 - 0.68) | 0.1573 |
| 4 | ISOA28_wt_vs_wt_I | S2 | 5 | 0.02 (-0.14 - 0.18) | 0.7661 | 0.69 (0.61 - 0.78) | 0.0000 | 0.32 (0.23 - 0.41) | 3e-04 |
| 5 | ISOA28_wt_vs_wt_II | S2 | 6 | 0.11 (-0.07 - 0.29) | 0.2195 | 0.48 (0.38 - 0.58) | 0.6589 | 0.45 (0.35 - 0.55) | 0.3392 |
| 6 | ISOO11_wt_vs_wt_I | S2 | 7 | 0.07 (-0.1 - 0.25) | 0.4239 | 0.59 (0.49 - 0.68) | 0.0733 | 0.66 (0.56 - 0.75) | 0.0013 |
| 7 | ISOO11_wt_vs_wt_II | S2 | 8 | -0.15 (-0.32 - 0.03) | 0.1102 | 0.44 (0.33 - 0.54) | 0.2533 | 0.40 (0.31 - 0.50) | 0.0501 |
| 8 | A28_wt_vs_kd_F4 | S2 | 1 | 0.94 (0.87 - 0.98) | 0.0000 | 0.00 (0 - 0) | 0.0334 | 0.75 (0.66 - 0.82) | 0.0000 |
| 9 | A28_wt_vs_kd_F5 | S3 | 2 | 0.75 (0.62 - 0.86) | 0.0000 | 0.32 (0.14 - 0.57) | 0.1492 | 0.45 (0.35 - 0.55) | 0.2971 |
| 10 | A28_wt_vs_kd_F13 | S3 | 3 | 0.13 (-0.05 - 0.31) | 0.1473 | 0.48 (0.38 - 0.58) | 0.6915 | 0.50 (0.40 - 0.59) | 0.9352 |
| 11 | O11_wt_vs_kd_F4 | S3 | 4 | 0.92 (0.83 - 0.97) | 0.0000 | 0.78 (0.35 - 0.99) | 0.1985 | 0.35 (0.25 - 0.45) | 0.0034 |
| 12 | O11_wt_vs_kd_F5 | S3 | 5 | 0.71 (0.57 - 0.82) | 0.0000 | 0.65 (0.44 - 0.83) | 0.1609 | 0.57 (0.47 - 0.66) | 0.1749 |
| 13 | O11_wt_vs_kd_F13 | S3 | 6 | -0.05 (-0.21 - 0.12) | 0.6008 | 0.35 (0.26 - 0.44) | 0.0012 | 0.67 (0.57 - 0.75) | 7e-04 |
| 14 | ISOA28_kd_vs_kd_F8 | S3 | 7 | 0.51 (0.34 - 0.65) | 0.0000 | 0.48 (0.33 - 0.64) | 0.8103 | 0.58 (0.49 - 0.67) | 0.0970 |
| 15 | ISOO11_kd_vs_kd_F8 | S3 | 8 | 0.46 (0.28 - 0.61) | 0.0000 | 0.60 (0.45 - 0.74) | 0.1787 | 0.65 (0.55 - 0.74) | 0.0041 |
| 16 | A28_wt_vs_gfr_F10 | S4 | 1 | 0.88 (0.78 - 0.95) | 0.0000 | 0.29 (0.06 - 0.64) | 0.2447 | 0.59 (0.50 - 0.68) | 0.0599 |
| 17 | O11_wt_vs_gfr_F10 | S4 | 2 | 0.92 (0.83 - 0.97) | 0.0000 | 0.56 (0.18 - 0.91) | 0.7770 | 0.38 (0.28 - 0.48) | 0.0157 |
| 18 | A28wtxA28et_control | S4 | 3 | 0.18 (0 - 0.35) | 0.0523 | 0.45 (0.34 - 0.56) | 0.3547 | 0.74 (0.65 - 0.81) | 0.0000 |
| 19 | O11wtxO11et_control | S4 | 4 | -0.02 (-0.18 - 0.15) | 0.8262 | 0.66 (0.57 - 0.75) | 5e-04 | 0.52 (0.42 - 0.63) | 0.6406 |
| 20 | A28wtxA28ps_control | S4 | 5 | -0.03 (-0.21 - 0.15) | 0.7468 | 0.53 (0.43 - 0.62) | 0.5538 | 0.68 (0.59 - 0.77) | 3e-04 |
| 21 | O11wtxO11ps_control | S4 | 6 | 0.02 (-0.15 - 0.18) | 0.8272 | 0.67 (0.58 - 0.75) | 3e-04 | 0.73 (0.62 - 0.81) | 0.0000 |
| 22  **Table S5. Estimation of SII (Sexual Isolation Index), pM (‘Male preference’) and pF (‘Female preference’**). pM and pF reflect the bias in the mating order. No bias corresponds to a value of 0.5. The *p* values are computed from likelihood ratio test, the null hypothesis being random mating (for e SII) and no bias in the mating order (for pM and pF). | A28wtxO11ps | S4 | 7 | 0.92 (0.83 - 0.98) | 0.0000 | 0.00 (0 - 0) | 0.0098 | 0.51 (0.42 - 0.61) | 0.7707 |
