# Supplementary material for: Symbiont-Driven Male Mating Success in the Neotropical Drosophila paulistorum Superspecies
Source: Behav Genet. 2018 Nov 19;49(1):83–98. doi: 10.1007/s10519-018-9937-8 (PMC6327003; doi:10.1007/s10519-018-9937-8)
Supplement: Supplementary file 11 — Supplementary material 11 (DOCX 105 KB) [file 10519_2018_9937_MOESM11_ESM.docx]

| No | Compound name | Retention time (min) | Retention index | Specificity |
| --- | --- | --- | --- | --- |
| 1 | 11-Docosenyl-acetate (C_24_H_46_O_2_) | 18.816 | 25.79 | male-specific |
| 2 | 19-Triacontenyl-acetate (C_32_H_62_O_2_) | 28.670 | 33.86 | male-specific |
| 3 | 2-Methyl-triacontane (C_31_H_64_) | 24.465 | 30.63 | male-specific |
| 4 | a di-unsaturated acetate (C_32_H_60_O_2_) | 28.175 | 33.54 | male-specific |
| 5 | Methyl-(Z)-9-tetradecanoate (C_15_H_28_O_2_) | 6.5920 | 17.13 | none |
| 6 | Ethyl-9-tetradecenoate (C_16_H_30_O_2_) | 7.5420 | 17.80 | none |
| 7 | MethylC28 | 22.228 | 28.62 | none |
| 8 | MethylC29 | 23.349 | 29.61 | none |
| 9* | unknown compound | 26.797 | 32.60 | none |
| 10 | C31:1 | 24.619 | 30.78 | none |
| 11 | C33:1A | 26.577 | 32.43 | none |
| 12 | C33:1B | 26.683 | 32.51 | none |
| 13* | C33:2A+unknown | 26.795 | 32.60 | none |
| 14 | C33:2B | 26.915 | 32.69 | none |
| 15 | C33:2C | 27.038 | 32.78 | none |
| 16 | C33:2D | 27.173 | 32.88 | none |
| 17 | C33:2E | 27.320 | 32.99 | none |
| 18 | C35:1A | 29.581 | 34.37 | none |
| 19 | C35:1B | 29.736 | 34.45 | none |
| 20 | C35:2A | 29.922 | 34.55 | none |
| 21 | C35:2B | 30.069 | 34.63 | none |
| 22 | C35:2C | 30.250 | 34.73 | none |
| 23 | C35:2D | 30.409 | 34.81 | none |
| 24 | C35:2E | 30.590 | 34.91 | none |
| 25 | C37:2A | 34.569 | 36.57 | none |
| 26 | C37:2B | 34.783 | 36.64 | none |
| 27 | C37:2C | 35.017 | 36.73 | none |

**Table S6. Full list of compounds from GC/MS analysis of *D. paulistorum* males.** Retention times determined *via* gas chromatography coupled to mass-spectrometry are given in minutes for each of the 27 compounds. Retention indices were calculated based on an external alkane standard. Asterisks mark two additional compounds that were not included in the previous studies by (Kim et al. 2004; Chao et al. 2010).
