# Supplementary material for: Symbiont-Driven Male Mating Success in the Neotropical Drosophila paulistorum Superspecies
Source: Behav Genet. 2018 Nov 19;49(1):83–98. doi: 10.1007/s10519-018-9937-8 (PMC6327003; doi:10.1007/s10519-018-9937-8)
Supplement: Supplementary file 12 — Supplementary material 12 (PDF 243 KB) [file 10519_2018_9937_MOESM12_ESM.pdf]

```
######################################################################
##
#
# Assortative mating in non-replacement mating assays
#
# Implementation of the statistical model
# Arnaud Le Rouzic 2017 lerouzic@egce.cnrs-gif.fr
#
# To be published along with Schneider et al.,
# "Influence of mutualistic Wolbachia on sexual isolation in
# the neotropical Drosophila paulistorum superspecies".
#
# This script is distributed under the WTFPL version 2.
#
######################################################################
##

# Summary:
#
# The model is intended to estimate by maximum likelihood the
parameters
# of a mating assay with limited replacement.
#
# An experiment typically consist in 4N individuals (2N males and 2N
# females) from two distinct origins (say, A and B). The parameter of
# interest is the assortative mating rate h, which can be positive
# or negative (disassortative mating). Individuals mate sequentially,
# and might choose only among non-mated partners (the order of mating
# restricts the choice).
#
# Parameters are:
# h: assortative mating rate. (between -1 (full disassortative mating)
and
#    1 (full assortative mating)
# pM: preference for males from the first population (quantifies how
much
#    any kind of females prefer males from the first population).
#    0: only males of population B are chosen,
#    1: only males from population A,
#    0.5: no proference
# pF: bias in mating speed of females.
#     0: females from population B mate first
#     1: females from population A mate first
#     0.5: random order of mating
# remating: the probability for a male or a female already mated to
mate again.
#
# Dataset:
# The data consists in a list of experiments.
# An experiment is itself a list containing three named fields:
```

```
#   $N: scalar, number of males/females from each population.
#   $females: vector of a 2-level factor, ordered set of mated females
#   $males:  vector (same factor levels as above, ordered set of mated
males.
#
# Key functions:
#   * mle.model2, mle.model2b, mle.model2c: maximum-likelihood
estimates
#   * summary.model2b, summary.model2c: gives a summary table with,
for
#     each parameter, the estimate, confidence interval, and
#     likelihood-ratio test.
#   model2 is a 3-parameter model, with remating=0.
#   model2b is a 4-parameter model, estimating remating
#   model2c is a 3-parameter model, remating being fixed to 0.05$
#
# Implementation details:
#  * negative and positive assortative mating are treated separately.
#    The likelihood of both models converge when h = 0; making it
#    possible to estimate h within the same framework and to hide the
#    twin-model setup to the user.
#  * parameters are transformed before fitting. Probabilities (pM and
pF)
#    are logit-transformed, and h is scaled in a similar way
#    (-1;1)->(-Inf,Inf). (see function logit2 below). They are then
#    reversed-transformed in the summary functions.
#  * To make the fit possible when probabilities tend to extreme
values, t
#    he likelihood is artificially deflated when probability
parameters
#    tend to extreme values (0 or 1). The threshold from which the
#    likelihood function changes is called "precision" in the
probaPairs()
#    function. If the likelihood converges to such a limit, the
estimated
#    confidence intervals are meaningless for this paramater
#  * The model is poorly defined (parameters are not separable) in
#    extreme cases (e.g: pM = pF = 1 makes h meaningless). Such
datasets
#    may generate convergence issues.



library(stats4)

algo <- 1    # Possibility to compare several variants of the
algorithm, not used so far.

############### Helper functions (variable transformations)
############
```

```
# logit (logistic transformation) maps p (0,1) to x (-Inf, Inf)
# unlogit does the inverse transformation
# logit2 maps h (-1, 1) to y (-Inf, Inf)
# unlogit2 does the inverse transformation
logit <- function(p)  log(p/(1-p))
unlogit <- function(x) 1/(1+exp(-x))
logit2 <- function(h) log((1+h)/(1-h))
unlogit2 <- function(y) { ey <- exp(y); ifelse(y==Inf, 1, (ey-1)/(ey
+1)) }

# This detects the transformation to apply from the names of the
variables
untransform.u <- function(x, name=NULL, prefix="un") {
    if (is.null(name)) name <- names(x)
    ss <- strsplit(name, split="\\.")[[1]]
    FUN.name <- ss[1]
    var.name <- ss[2] # Assuming only one dot
    FUN <- try(get(paste0(prefix, FUN.name)))
    ans <- NA
    if (class(FUN) != "try-error") ans <- FUN(x)
    names(ans) <- var.name
    ans
}
untransform <- function(vx, vname=NULL) {
    sapply(seq_along(vx), function(i) untransform.u(vx[i], vname[i]))
}

################## Probability calculations (probabilistic model)
######

probaCore2 <- function(f.male, f.female, p.male, p.female, h,
remating=0.5) {
    # f.male: frequency of males from population A
    # f.female: frequency of females from population A
    # p.male: advantage of males from population A in the mating
process (female preference)
    # p.female: advantage of females from population A (speed of
mating)
    # h: rate of homogamy
    # remating: rate of remating.

    if(f.male < 0) f.male <- 0
    if (f.female < 0) f.female <- 0
    if(f.male > 1) f.male <- 1
    if (f.female > 1) f.female <- 1

    f.male <- f.male - remating*(f.male - 0.5)
    f.female <- f.female - 0.001*(f.female - 0.5)
```

```
    if (h >= 0) return(probaCore2.hpos(f.male, f.female, p.male,
p.female, h))
    else return(probaCore2.hneg(f.male, f.female, p.male, p.female,
h))
}

probaCore2.hpos <- function(f.male, f.female, p.male, p.female, h) {
    stopifnot( h >= 0)
    # a bit of optimization
    h1 <- 1-h
    g.male <- 1-f.male
    g.female <- 1-f.female
    q.male <- 1-p.male
    q.female <- 1-p.female
    p.male.h1 <- p.male*h1
    f.male2 <- 2*f.male-1

    K1 <- p.female * f.female + q.female * g.female
    K2 <- g.male + (h+p.male.h1)*f.male2
    K3 <- g.male +    p.male.h1 *f.male2

    K1K2 <- K1*K2
    K1K3 <- K1*K3

    ans <- c(
        p.female * f.female * f.male * (h+p.male*(h1)) / K1K2,
        p.female * f.female * q.male * g.male * h1 / K1K2,
        q.female * g.female * f.male * p.male * h1 / K1K3,
        q.female * g.female * g.male * (1 - p.male * h1) / K1K3)
    # Codes stand for "population of female"-"population of male"
    names(ans) <- c("A-A","A-B","B-A","B-B")

    if (abs(sum(ans) - 1.0) > 1e-6) browser()  # If it does not add up
to 1, we have a problem

    return(ans)
}

probaCore2.hneg <- function(f.male, f.female, p.male, p.female, h) {
    stopifnot( h <= 0)
    # a bit of optimization
    h1 <- 1+h
    g.male <- 1-f.male
    g.female <- 1-f.female
    q.male <- 1-p.male
    q.female <- 1-p.female
    p.male.h1 <- p.male*h1

    K1 <- p.female * f.female + q.female * g.female
    K4 <- g.male*(1-p.male.h1)+f.male*p.male.h1
```

```
    K5 <- f.male*(p.male.h1-h)+g.male*(1+h-p.male.h1)

    K1K4 <- K1*K4
    K1K5 <- K1*K5

    ans <- c(
        p.female * f.female * f.male * p.male * h1 / K1K4,
        p.female * f.female * g.male * (1- h1*p.male) / K1K4,
        q.female * g.female * f.male * (p.male * h1 - h) / K1K5,
        q.female * g.female * g.male * (1 + h - p.male * h1) / K1K5)
    # Codes stand for "population of female"-"population of male"
    names(ans) <- c("A-A","A-B","B-A","B-B")

    if (abs(sum(ans) - 1.0) > 1e-6) browser()

    return(ans)
}

probaPairs <- function(males, females, N, logit.pM, logit.pF,
logit2.h, logit.remating=-Inf, check=TRUE, precision=1e-3) {
    # males and females are vectors of factors
    #  = ordered series of coupled males and females
    # pM, pF, h, and remating are the parameters to optimize
    # N is the number of males and females from each pop in the
experiment.

    if (!is.factor(males)) {
        males <- factor(males, levels=sort(unique(males)))
    }
    if (!is.factor(females)) {
        females <- factor(females, levels=sort(unique(females)))
    }

    if (check) {
        stopifnot(N > 0)
        stopifnot(length(males) > 0, length(females) > 0)
        stopifnot(length(levels(males)) == length(levels(females)))
        stopifnot(all(levels(males) == levels(females)))
        stopifnot(length(levels(males))==2)
    }
    # pre-computation
    llA <- levels(males)[1] # first factor level (== population A),
Same as for females
    pM <- unlogit(logit.pM)
    pF <- unlogit(logit.pF)
    h <- unlogit2(logit2.h)
    remating <- unlogit(logit.remating)

    mmllA <- males==llA
    ffllA <- females==llA
```

```
    # Calculation of the biases due to the change in frequencies
    fM <- (N-c(0,cumsum(mmllA)[-length(males)]))/(2*N-0:
(length(males)-1))
    fF <- (N-c(0,cumsum(ffllA)[-length(females)]))/(2*N-0:
(length(females)-1))

    obs <- paste(ifelse(ffllA, "A", "B"), ifelse(mmllA, "A", "B"),
sep="-")

    if (algo == 1) {
    probs <-
        mapply(obs, fM, fF, FUN=function(oo, fm, ff) {
            frqs <- probaCore2(fm, ff, pM, pF, h, remating)
            frqs[oo]
        })
    }
    probs <- unlist(probs)
    # if (any(probs==0)) browser()

    # Penalties for extreme frequency values (helping convergence?)
    penalty <- 1
    if (pM < precision) penalty <- penalty * pM
    if (pF < precision) penalty <- penalty * pF
    if (remating < precision) penalty <- penalty * remating
    if (pM > 1-precision) penalty <- penalty * (1-pM)
    if (pF > 1-precision) penalty <- penalty * (1-pF)
    if (remating > 1-precision) penalty <- penalty * (1-remating)

    probs <- probs * penalty

    return(prod(probs))
}

probaExperiment <- function(data, logit.pM, logit.pF, logit2.h,
logit.remating=-Inf, check=TRUE, log=TRUE) {
    is.dataOK <- function(dd) {
        stopifnot(is.list(dd))
        stopifnot(names(dd) %in% c("N","males","females"))
    }

    if (check) {
        stopifnot(is.list(data))
        invisible(sapply(data, is.dataOK))
    }

    ans <- sapply(data, function(dd) {
            probaPairs(males=dd$males, females=dd$females, N=dd$N,
            logit.pM=logit.pM, logit.pF=logit.pF, logit2.h=logit2.h,
logit.remating=logit.remating, check=check)
```

```
        })
    if (log) {
        return(sum(log(ans)))
    } else {
        return(prod(ans))
    }
}

############# Monte-Carlo simulations, for testing purposes
############

simul.model2 <- function(n, N, pM=0.5, pF=0.5, h=0, remating=0) {
    simul.model2.uniq <- function(N, pM, pF, h, remating) {
        ans <- list(N=N)

        for (nn in 1:(2*N)) { # We don't care about efficiency here.

            femalesA.remain <- N - sum(ans$females == "A")
            malesA.remain <- N - sum(ans$males == "A")

            # If remating is allowed, the model behaves strangely ->
not important?
            # A trick is necessary to avoid negative frequencies
            freqMales <- max(malesA.remain/(2*N-nn+1), 0)
            freqFemales <- max(femalesA.remain/(2*N-nn+1),0)
            prb.pairs <- probaCore2(freqMales, freqFemales, pM, pF, h,
remating)

            andthewinneris <- sample(names(prb.pairs), 1,
prob=prb.pairs)

            sp <- strsplit(andthewinneris, split="-")[[1]]
            ans$females <- c(ans$females, sp[1])
            ans$males <- c(ans$males, sp[2])
        }
        return(ans)
    }

    replicate(n, simul.model2.uniq(N=N, pM=pM, pF=pF, h=h,
remating=remating), simplify=FALSE)
}

############################## Model fitting
##############################

mle.model2 <- function(data, confint=TRUE, profile=FALSE, check=TRUE)
{
    # 3 parameters, without remating
    minuslogL2 <- function(logit.pM, logit.pF, logit2.h)
        -probaExperiment(data=data, logit.pM=logit.pM,
```

```
logit.pF=logit.pF, logit2.h=logit2.h, log=TRUE, check=check)
    ans <- mle(minuslogL2, start=list(logit.pM=0, logit.pF=0,
logit2.h=0))
     list(model=ans, confint=if(confint) confint(ans) else NA,
profile=if(profile) profile(ans) else NA,
        modsel=list(
            wo.h=mle(minuslogL2, start=list(logit.pM=0, logit.pF=0),
fixed=list(logit2.h=0)),
            wo.pM=mle(minuslogL2, start=list(logit2.h=0, logit.pF=0),
fixed=list(logit.pM=0)),
            wo.pF=mle(minuslogL2, start=list(logit2.h=0, logit.pM=0),
fixed=list(logit.pF=0)))
    )
}

mle.model2b <- function(data, confint=TRUE, profile=FALSE, check=TRUE)
{
    # 4 parameters, including remating
    minuslogL2b <- function(logit.pM, logit.pF, logit2.h,
logit.remating)
        -probaExperiment(data=data, logit.pM=logit.pM,
logit.pF=logit.pF, logit2.h=logit2.h, logit.remating=logit.remating,
log=TRUE, check=check)
    ans <- mle(minuslogL2b, start=list(logit.pM=0, logit.pF=0,
logit2.h=0, logit.remating=-5))
    list(model=ans, confint=if(confint) confint(ans) else NA,
profile=if(profile) profile(ans) else NA,
        modsel=list(
            wo.h=mle(minuslogL2b, start=list(logit.pM=0, logit.pF=0,
logit.remating=-5), fixed=list(logit2.h=0)),
            wo.pM=mle(minuslogL2b, start=list(logit2.h=0, logit.pF=0,
logit.remating=-5), fixed=list(logit.pM=0)),
            wo.pF=mle(minuslogL2b, start=list(logit2.h=0, logit.pM=0,
logit.remating=-5), fixed=list(logit.pF=0)))
    )
}

summary.model2b <- function(data, check=TRUE, ...) {
    mod <- mle.model2b(data, check=check, ...)
    pp <- c("h", "pM", "pF", "remating")
    transfpp <- c("logit2", "logit", "logit", "logit")
    ans <- do.call(rbind, lapply(pp, function(ppp) {
            mylogLik <- function(x) if (is.null(x)) NA else logLik(x)
            logLikFull <- mylogLik(mod$model)
            logLikAlt <- mylogLik(mod$modsel[[paste0("wo.",ppp)]])
            c(untransform(coef(mod$model)[paste0(transfpp, ppp)]),
untransform(mod$confint[paste0(transfpp, ppp),]), logLikFull,
logLikAlt, -2*(logLikAlt-logLikFull), 1-pchisq(-2*(logLikAlt-
logLikFull), df=1))
         }))
```

```
    colnames(ans) <- c("Estimate", "CI-2.5%", "CI-97.5%",
"logLik(H1)", "logLik(H0)", "LR test", "p (Chisq)")
    rownames(ans) <- pp
    ans
}


mle.model2c <- function(data, confint=TRUE, profile=FALSE, check=TRUE,
start=list(logit.pM=0, logit.pF=0, logit2.h=0),
logit.remating=logit(0.05)) {
    # 3 parameters, remating fixed at 5%
    minuslogL2c <- function(logit.pM, logit.pF, logit2.h)
        -probaExperiment(data=data, logit.pM=logit.pM,
logit.pF=logit.pF, logit2.h=logit2.h, logit.remating=logit.remating,
log=TRUE, check=check)
    ans <- mle(minuslogL2c, start=start)
    list(model=ans, confint=if(confint) confint(ans) else NA,
profile=if(profile) profile(ans) else NA,
        modsel=list(
            wo.h=mle(minuslogL2c, start=list(logit.pM=0, logit.pF=0),
fixed=list(logit2.h=0)),
            wo.pM=mle(minuslogL2c, start=list(logit2.h=0, logit.pF=0),
fixed=list(logit.pM=0)),
            wo.pF=mle(minuslogL2c, start=list(logit2.h=0, logit.pM=0),
fixed=list(logit.pF=0)))
    )
}

summary.model2c <- function(data, check=TRUE, ...) {
    mod <- mle.model2c(data, check=check, ...)
    pp <- c("h", "pM", "pF")
    transfpp <- c("logit2", "logit", "logit")
    ans <- do.call(rbind, lapply(pp, function(ppp) {
            mylogLik <- function(x) if (is.null(x)) NA else logLik(x)
            logLikFull <- mylogLik(mod$model)
            logLikAlt <- mylogLik(mod$modsel[[paste0("wo.",ppp)]])
            wpp <- which(grepl(names(coef(mod$model)),
pattern=paste0(ppp,"$")))
            c(untransform(coef(mod$model)[wpp]), if(!is.na(mod
$confint)) untransform(mod$confint[wpp,], rep(rownames(mod$confint)
[wpp], 2)) else c(NA,NA), logLikFull, logLikAlt, -2*(logLikAlt-
logLikFull), 1-pchisq(-2*(logLikAlt-logLikFull), df=1))
         }))
    colnames(ans) <- c("Estimate", "CI-2.5%", "CI-97.5%",
"logLik(H1)", "logLik(H0)", "LR test", "p (Chisq)")
    rownames(ans) <- pp
    ans
}
```

```
##################################
# Script to analyse the data
# Aurelie Hua-Van: aurelie.hua-van@egce.cnrs-gif.fr

# Run this script to regenerate all the data presented in Table S2-S5,
Figure 3 and Figure S3 in
# Schneider et al.,
# "Influence of mutualistic Wolbachia on sexual isolation in
# the neotropical Drosophila paulistorum superspecies".


#### How to proceed ###########################################
# Please set the working directory ( setwd('Current/directory') ) to
current directory, if necessary (or to directory containing the script
model2.R and Data folder)
# Copy and paste all this file in the terminal, or:
# Paste the part 0 (various functions)
# Paste the part 1 to run the model with 3 different remating rates on
the different assays (you can choose the male remating rate)
# Paste the part 2 to run the model for individual replicates with
remating rate of 0.5 (you can choose the male remating rate)
# Paste part 3 for calculation of uncorrected individual and per assay
SII
# Paste part 4 to compile all the results in a huge file
(Results.stat.txt)
### Part 4 needs all previous parts to have been run (and part 2 run
with remating.rate of 0.5)
##############################################################

###################################################################
########## Part 0 - Model, functions and variables ###############
###################################################################
source("model2.R")

# List of files to read (in a Data directory in the working directory)
fileliste <-list.files(path=paste(getwd(),'/Data',sep=''),
pattern='.txt')

# Prefix of folder for data run with different remating rate
resfolder.prefix='Results_'

# Function that transforms the data table into a list of $5 * 3 items
(number of fly of each type (nrow/2), males, and females in mating
order)
df2list <- function(df) {
    mxrep <- ncol(df)/2 # number of replicas
    ans <- list()
    for (i in 1:mxrep) {
        ans[[i]] <- list(
            N=nrow(df)/2,
```

```
            males = df[,paste0("rep", i, ".M")],
            females = df[,paste0("rep", i, ".F")])
    }
    ans
}

# Function that extracts the name of the cross from the name of the
file
# File names with the form TableAssay_Cross.txt
spl <- function(x) {
        expe <-as.character(strsplit(as.character(x),'.txt')[[1]][1])
        assay <-paste0(as.character(strsplit(as.character(x),'_')[[1]]
[1]),'_')
    return(gsub(assay,'',expe))
}

# Function that extracts the table and the assay numbers from the name
of the file
GetAssayNumber <- function(x) {
        assay <-as.character(strsplit(as.character(x),'_')[[1]][1])
        expe <-strsplit(as.character(assay),'A')[[1]]
        return(unlist(expe))
}

# Function that calculates the SII based on the number of each kind of
couples
# order in x : AA, AB, BA, BB (females are first)
calcSII <- function(x) {
     return((x[1]+x[4]-x[2]- x[3])/sum(x))
}

########################## End of part 0
####################################

#######################################################################
### part 1 - Estimation of the parameters (h, pM, pF) per assay ######
#######################################################################
# Calculate, h, CI, Likelihood Ratio Test and pvalue, write the
results in a file, for each file (experiment).
# Results will be stored in a folder "Results_XX" where XX correspond
to the remating rate of the male (by default: 0.5 )
# If the folder already exist, the content may be erased.
remating.rate = c(0.05,0.5,0.8) # put here the male remating rates you
want to test (remating of female is fixed to 0.001 (0 generates
problem during log transformation) in the model2.R script (#126))

for (rr in remating.rate){
        dir.create(paste0(resfolder.prefix,as.character(rr)))
    for (filename in fileliste){
            df1 <-read.table(file=paste(getwd(),'/
```

```
Data/',filename,sep=''), header=TRUE)
        data2 <- df2list(df1)
        res <- summary.model2c(data2, logit.remating=logit(rr))
    write.table(res,file=paste0(resfolder.prefix,as.character(rr),
    '/',filename,'.results.txt'),quote=FALSE,row.names=TRUE, sep='\t')
    }
}
########################## End of part 1
####################################

######################################################################
############
#### part 2 - Estimation of the parameters for individual
replicas###############
######################################################################
############
# Calculate, h, Likelihood Ratio Test and pvalue, write the results in
a file, for each replica (CI is disabled)
# Results will be stored in a folder "Results.XX.ind" where XX
correspond to the remating rate of the male (by default: 0.5 )
# If the folder already exist, the content may be erased.
remating.rate = 0.5 # change here the remating of the males (remating
of female is fixed to 0.001)
dir.create(paste0('Results.',as.character(remating.rate),'.ind'))
h <- c()
for (filename in fileliste){
        df1 <-read.table(file=paste(getwd(),'/Data/',filename,sep=''),
header=TRUE)
    data2 <- df2list(df1)
    for (i in 1:length(data2)){
        res <- summary.model2c(data2[i],
logit.remating=logit(remating.rate), confint= FALSE)

write.table(res,file=paste0('Results.',as.character(remating.rate),'.i
nd','/',filename,'.',i,'.results.txt'),quote=FALSE,row.names=TRUE,
sep='\t')
        h <- c(h,res[1,1])
    }
}
mat <- as.data.frame(matrix(h, ncol=5, nrow=22, byrow=TRUE))
colnames(mat)<-c("erep1","erep2","erep3","erep4","erep5")
mat$Cross <- lapply(fileliste, spl)
Tab <- lapply(fileliste, GetAssayNumber)
TA <- as.data.frame(matrix(unlist(Tab),ncol=2,nrow=22,byrow=TRUE))
mat$Table <- TA$V1
mat$Assay <- as.numeric(TA$V2)

AS <- read.table('Assays_summary.txt',sep='\t',header=TRUE)
mat2 <-merge(AS,mat,by=c('Cross','Table','Assay'))
write.table(mat2,
```

```
paste0('Results.eSII.ind.',remating.rate,'.txt'),quote=FALSE,
row.names=FALSE,col.names=TRUE, sep='\t')

########################## End of part 2
####################################

######################################################################
##########
#### part 3 -  SIIs calculated by the old method
#############################
######################################################################
##########
# Counts the number of the different crosses, calculate SII + SE, and
Fisher test p-value (old method) --> Results.SII.txt
msii <- matrix(NA, nrow=length(fileliste), ncol= 20)
colnames(msii)<-c("Cross", "Table","Assay","SII", "SE","SII +/-
SE","Fisher p
value","rep1","rep2","rep3","rep4","rep5","AA","AB","BA","BB","Females
A","FemalesB","MalesA","MalesB")
for (n in 1:length(fileliste)){
        vec <- c()
        df1 <-read.table(file=paste(getwd(),'/Data/',
fileliste[n],sep=''), header=TRUE)
    mxrep <- ncol(df1)/2
    AA<-0
    AB<-0
    BA<-0
    BB<-0
    rep <-c()
    for (i in 1:mxrep) {
        males = df1[,paste0("rep", i, ".M")]
        females = df1[,paste0("rep", i, ".F")]
        aa <- length(males[males =='A' & females =='A'])
        ab <- length(males[males =='B' & females =='A']) # AB : female
A and male B
        ba <- length(males[males =='A' & females =='B']) # BA : female
B and male A
        bb <- length(males[males =='B' & females =='B'])
        AA <- AA+aa
        AB <- AB+ab
        BA <- BA+ba
        BB <- BB+bb
        rep <-c(rep,calcSII(c(aa,ab,ba,bb)))
        if (aa+ab !=ba+bb) print(c(aa,ab,ba,cc))
        }
    SII <- calcSII(c(AA,AB,BA,BB))
    SE <- sqrt((1-SII*SII)/120)
    p <-fisher.test(matrix(c(AA,AB,BA,BB),ncol=2,nrow=2))$p.value
    Cross <- spl(fileliste[n])
    Table <- GetAssayNumber(fileliste[n])[1]
```

```
    Assay <- as.numeric(GetAssayNumber(fileliste[n])[2])
    vec <-c(Cross,Table,Assay,SII,SE,paste(round(SII,2),' +/-
',round(SE,2),sep=''),p,rep,AA,AB,BA,BB,AA+AB,BA+BB,AA+BA,AB+BB)
    msii[n,] <- vec
}
msii2 <- as.data.frame(msii)
AS <- read.table('Assays_summary.txt',sep='\t',header=TRUE)
tot <-merge(AS,msii2,by=c('Cross','Table','Assay'))
tot <- tot[order(tot$Table,tot$Assay),]
write.table(msii2,'Results.SII.txt',sep='\t',col.names=TRUE,
quote=FALSE, row.names=FALSE )
########################## End of part 3
####################################


######################################################################
################################
#### part 4 - Compilation of the SIIs and the estimated SII for the
various remating rates
#### (per replica and per assay)
######################################################################
################################
# Compile SII and estimates of h, PF, pM for the various remating
rates --> Results.stat.txt
Res.folder <- dir(path='.', pattern= resfolder.prefix, include.dirs =
TRUE)
val <- c('', 'CI.2.5','CI.97.5','pval')
estimate <- c('_h','_pM','_pF')
nom <- outer(val , estimate, FUN=paste0)
m <- matrix(NA, nrow=length(fileliste), ncol=
length(Res.folder)*length(nom))
for (n in 1:length(fileliste)){
        vec <- c()
        namevec<-c()
                for (fold in Res.folder){
                remating <- strsplit(fold, '_')[[1]][2]
            df <-read.table(file=paste(getwd(),fold,
paste0(fileliste[n],'.results.txt'),sep='/'), header=TRUE, sep='\t')
            # h, h.CI.2.5,h.CI.97.5,h.pval,pM,pM.CI.2.5,pM.CI.
97.5,pM.pval,pF,pF.CI.2.5,pF.CI.97.5,pF.pval
            vec <-
c(vec,df[1,c(1,2,3,7)],df[2,c(1,2,3,7)],df[3,c(1,2,3,7)])
            namevec <- c(namevec, paste(remating, nom,sep='_')  )
            }
        est <- unlist(vec)
        names(est) <-unlist(namevec)
        m[n,] <-est
}
m <-as.data.frame(m)
colnames(m)=names(est)
```

```
m$Cross <- unlist(lapply(fileliste,spl))

sii<- read.table('Results.SII.txt',sep='\t',header=TRUE)
esii<- read.table('Results.eSII.ind.0.5.txt',sep='\t',header=TRUE)
sii2<-merge(sii,esii,by=intersect(colnames(sii),colnames(esii)))
m2 <-merge(sii2,m,by='Cross')
m2 <- m2[order(m2$Table,m2$Assay),]
write.table(m2,'Results.stat.txt',sep='\t',col.names=TRUE,
quote=FALSE, row.names=FALSE )
```

This folder contains :

1- Data folder : 22 files corresponding to the 22 assays (raw data)
- header line : the replica number (rep1 to rep5) and the sex of the flies (.M or .F)
- rows are ordered by mating order (row 1 = first mating, row 24 = last mating)
- strains are computed as A or B (correspondence to strains is indicated in the second comment line (#)

2- Assays_Summary.txt : Characteristics of the 22 assays (strains, treatment, etc..)

3- model2.R : the R script encoding the model

4- EstimateSII.R : the R script that runs the model on the data
0- Functions and variables needed
1- Estimation of the parameters on the assays (several replicas) (different runs can be done with different male remating rates)
2- Estimation of the parameters on the individual replicas (different runs can be done with different male remating rates)
3- Calculate the old-method SII for the different assays
4- Compile everything (Results.stat.txt)

All these allow to generate all the results presented in Tables S2-S5, in Figure 3 and in Figure S3.

Cross	Table	Assay	Type	Genotype1	Genotype2	Strain
AxO_control	S2	2	pool	wt	wt	A28.O11
AxA_control	S2	3	pool	wt	wt	A28
OxO_control	S2	4	pool	wt	wt	O11
ISOA28_wt_vs_wt_I	S2	5	iso	wt	wt	A28
ISOA28_wt_vs_wt_II	S2	6	iso	wt	wt	A28
ISOO11_wt_vs_wt_I	S2	7	iso	wt	wt	O11
ISOO11_wt_vs_wt_II	S2	8	iso	wt	wt	O11
A28_wt_vs_kd_F4	S3	1	pool	wt	kd	A28
A28_wt_vs_kd_F5	S3	2	pool	wt	kd	A28
A28_wt_vs_kd_F13	S3	3	pool	wt	kd	A28
O11_wt_vs_kd_F4	S3	4	pool	wt	kd	O11
O11_wt_vs_kd_F5	S3	5	pool	wt	kd	O11
O11_wt_vs_kd_F13	S3	6	pool	wt	kd	O11
ISOA28_kd_vs_kd_F8	S3	7	iso	kd	kd	A28
ISOO11_kd_vs_kd_F8	S3	8	iso	kd	kd	O11
A28_wt_vs_gfr_F10	S4	1	gfr	wt	gfr	A28
O11_wt_vs_gfr_F10	S4	2	gfr	wt	gfr	O11
A28wtxA28et_control	S4	3	et	wt	et	A28
O11wtxO11et_control	S4	4	et	wt	et	O11
A28wtxA28ps_control	S4	5	ps	wt	ps	A28
O11wtxO11ps_control	S4	6	ps	wt	ps	O11
A28wtxO11ps	S4	7	ps	wt	ps	A28.O11

Table S2 Assay 2
AxO; wt vs wt; AM=A, OR=B;

rep1.F	rep1.M	rep2.F	rep2.M	rep3.F	rep3.M	rep4.F	rep4.M	rep5.F	rep5.M
B	B	A	A	B	B	B	B	A	A
B	B	A	A	B	B	A	A	B	B
B	B	A	A	A	B	B	B	A	A
B	B	B	B	A	A	B	B	A	A
A	A	B	B	A	A	B	B	A	A
A	A	B	B	A	A	B	A	A	A
A	A	A	B	A	A	A	A	A	A
A	A	A	B	A	A	A	B	A	A
A	B	B	A	B	B	B	B	A	A
A	B	A	B	B	B	B	B	A	A
B	B	A	A	B	B	A	A	B	B
B	B	B	B	B	B	B	A	B	A
A	A	A	A	A	B	B	B	A	B
A	A	B	B	A	A	A	A	B	B
A	A	A	A	A	A	A	A	B	B
A	B	B	B	B	B	B	B	A	A
A	A	A	A	A	A	A	A	A	A
A	A	A	A	A	A	B	B	B	B
B	B	B	B	A	A	B	B	B	B
B	B	B	B	B	B	A	A	B	B
B	B	A	B	B	B	A	A	B	B
B	B	B	B	B	B	A	A	B	A
B	B	B	B	B	B	A	A	B	A
B	B	B	A	B	B	A	A	B	B

Table S2 Assay 3
AxA; wt vs wt;AM1=A AM2=B;

rep1.F	rep1.M	rep2.F	rep2.M	rep3.F	rep3.M	rep4.F	rep4.M	rep5.F	rep5.M
B	B	A	B	A	A	B	A	B	B
A	B	A	A	B	A	B	A	A	B
B	B	A	A	B	B	B	A	A	A
A	B	A	B	B	B	B	A	B	B
A	A	B	B	B	B	A	B	A	A
A	A	A	A	B	B	A	B	A	B
B	A	B	B	B	B	A	A	A	A
B	A	A	A	B	B	A	A	B	B
A	A	A	B	B	B	A	B	A	B
A	A	A	B	B	B	A	B	B	A
B	A	A	B	B	A	A	A	B	B
A	B	A	B	B	A	A	A	B	B
B	B	B	B	B	A	B	B	A	A
B	A	A	B	A	B	B	B	A	A
B	B	B	A	A	B	A	A	B	A
A	A	B	B	A	A	A	A	A	B
B	A	A	A	A	A	B	B	B	A
A	A	B	A	A	A	A	B	A	B
B	B	B	A	A	B	A	A	A	B
A	A	B	B	A	B	B	A	B	B
A	A	B	A	A	B	B	A	B	A
A	A	B	A	A	B	B	B	B	A
B	B	B	B	A	B	B	B	A	B
B	A	B	A	A	B	B	B	B	A

Table S2 Assay 4;
OxO; wt vs wt; OR1=A OR2=B;

rep1.F	rep1.M	rep2.F	rep2.M	rep3.F	rep3.M	rep4.F	rep4.M	rep5.F	rep5.M
A	A	A	A	B	B	A	A	B	B
A	A	B	A	A	A	A	A	B	B
A	A	B	A	A	A	B	B	B	A
A	A	A	B	A	A	B	B	B	A
A	B	A	A	A	A	A	A	B	B
A	A	B	A	B	A	B	A	B	A
A	B	A	A	B	A	A	B	B	A
A	A	A	B	B	A	B	B	B	A
B	A	A	B	B	B	B	A	B	A
A	B	A	A	A	B	B	A	B	B
A	B	A	B	B	B	B	A	B	B
A	B	A	B	B	B	A	B	A	A
A	A	A	A	B	B	A	B	A	A
B	B	A	B	A	A	A	B	A	B
B	A	A	A	B	B	A	B	A	B
B	A	B	A	A	B	B	B	A	A
B	A	B	A	A	B	B	B	A	A
B	A	B	A	B	B	A	B	A	B
B	A	B	A	A	B	A	B	A	B
B	B	B	B	A	A	A	B	B	A
B	A	B	A	A	A	B	A	A	A
B	A	B	A	B	B	B	A	A	B
B	A	B	B	B	A	A	B	A	B
B	A	B	B	A	A	B	B	A	B

Table S2 Assay 5
isoA28; iso-female 4 vs 5; 4=A; 5=B

rep1.F	rep1.M	rep2.F	rep2.M	rep3.F	rep3.M	rep4.F	rep4.M	rep5.F	rep5.M
B	A	A	B	B	B	B	B	B	A
A	B	A	A	B	B	B	B	B	A
A	A	A	A	A	A	B	A	B	B
B	B	A	B	B	A	B	A	A	A
A	A	B	A	A	B	B	A	A	A
A	B	B	A	B	A	B	A	A	A
A	A	B	B	B	B	B	A	B	B
A	A	B	B	A	A	A	A	B	B
A	B	B	A	B	B	B	A	B	A
A	A	B	A	B	A	B	A	B	A
A	B	B	A	A	B	A	B	A	A
B	A	A	B	A	B	B	A	A	A
B	A	A	A	B	A	B	A	A	A
B	B	B	B	B	A	A	A	B	B
B	B	B	B	B	A	A	A	B	A
B	A	B	A	B	A	A	A	A	A
B	A	A	B	A	A	A	A	A	A
B	A	A	A	A	B	B	B	B	B
B	A	A	A	B	A	A	A	B	B
B	B	B	A	A	B	A	B	B	A
B	B	A	B	A	B	A	A	A	B
A	A	A	B	A	B	A	B	A	B
A	A	B	B	A	A	A	B	A	A
A	A	A	B	A	A	A	A	A	B

# AA	35
# AB	25
# BA	37
# BB	23

Table S2 Assay 6
isoA28; iso-female 2 vs 5; 2=A; 5=B

rep1.F	rep1.M	rep2.F	rep2.M	rep3.F	rep3.M	rep4.F	rep4.M	rep5.F	rep5.M
A	A	B	B	A	A	A	B	A	B
B	B	A	A	B	B	A	B	A	A
A	A	B	A	B	B	A	A	B	A
B	B	B	A	B	A	A	A	A	B
B	A	A	B	B	A	B	A	B	B
B	A	B	B	B	B	B	A	B	B
A	B	A	A	B	B	A	B	B	A
A	B	A	A	B	A	A	B	B	A
B	A	A	A	B	B	B	B	B	B
B	B	A	B	A	B	B	B	B	B
A	A	A	B	A	A	B	A	A	A
A	A	A	B	A	B	B	A	A	A
A	B	A	A	B	A	B	A	A	A
A	B	B	B	B	B	B	A	B	B
A	A	B	B	B	B	B	B	B	B
A	A	B	A	B	A	A	B	B	A
A	B	B	A	A	B	A	A	A	B
A	B	B	B	A	A	A	A	B	A
B	B	B	B	A	A	A	A	A	B
B	B	A	A	A	A	B	B	B	A
B	B	B	B	A	A	A	B	A	A
B	B	A	B	A	A	A	B	A	A
B	A	B	B	A	B	B	B	A	B
B	A	A	B	A	B	B	A	A	A

# AA	31
# AB	29
# BA	27
# BB	33

Table S2 Assay 7;
O11xO11; iso-female 1 vs 8; 1=A; 8=B

rep1.F	rep1.M	rep2.F	rep2.M	rep3.F	rep3.M	rep4.F	rep4.M	rep5.F	rep5.M
B	B	A	A	A	A	A	A	A	A
B	B	A	A	A	A	A	A	A	A
A	B	A	B	A	B	B	A	B	B
B	A	A	B	B	A	B	A	B	A
B	A	A	A	B	A	A	B	B	A
A	B	A	A	B	A	A	B	B	B
A	A	B	B	A	B	B	B	B	B
A	A	B	B	A	B	A	A	A	A
B	B	A	A	B	B	B	A	A	A
A	A	B	A	A	A	A	B	A	B
B	B	B	A	B	A	B	A	A	B
A	A	A	B	A	B	A	B	B	B
A	A	A	B	B	B	A	B	B	B
A	A	B	B	A	A	A	A	A	A
A	B	A	A	B	A	A	A	A	A
A	B	A	B	A	B	B	B	B	B
A	A	B	B	A	B	B	A	A	B
A	A	B	A	B	A	B	B	B	A
B	A	A	A	B	B	A	A	A	A
B	A	B	A	A	A	A	A	B	B
B	B	B	A	A	A	B	A	B	B
B	B	B	B	B	A	B	A	A	B
B	B	B	B	B	A	B	B	B	A
B	B	B	B	B	B	B	B	A	A

Table S2 Assay 8
O11xO11; iso-female 4 vs 8; 4=A; 8=B

rep1.F	rep1.M	rep2.F	rep2.M	rep3.F	rep3.M	rep4.F	rep4.M	rep5.F	rep5.M
A	B	A	A	B	B	B	A	B	B
A	B	A	A	B	B	A	B	A	A
B	A	B	B	B	A	A	A	B	A
A	A	B	B	B	A	B	B	B	A
B	B	A	A	A	B	A	B	B	A
A	A	A	A	A	B	A	B	B	A
B	B	B	B	A	A	B	A	A	B
B	A	A	B	A	A	B	A	B	B
B	A	B	A	B	B	A	B	B	B
B	B	B	A	B	B	A	A	B	A
A	A	B	A	A	A	B	A	B	B
B	A	B	A	B	A	A	A	B	B
B	A	B	A	B	A	A	B	A	B
A	B	A	B	A	B	A	B	A	B
A	B	A	A	A	B	B	A	B	B
A	B	A	B	B	B	B	A	A	B
A	B	B	B	B	B	B	B	A	B
B	A	B	B	A	A	A	B	A	B
B	A	A	A	A	A	B	A	A	B
B	B	B	B	B	A	A	A	A	B
B	B	B	B	A	B	A	A	B	B
A	A	A	B	B	A	B	B	A	B
A	A	A	B	A	B	B	B	A	B
A	A	A	B	A	B	B	B	A	A

Table S3 Assay 1
A28; wt vs kd; wt=A, kd=B

rep1.F	rep1.M	rep2.F	rep2.M	rep3.F	rep3.M	rep4.F	rep4.M	rep5.F	rep5.M
A	A	B	B	A	A	B	B	A	A
A	A	B	B	A	A	A	A	A	A
A	A	A	A	A	A	A	A	A	A
B	B	A	A	B	B	A	A	A	A
B	B	A	A	A	A	A	A	A	A
B	B	A	A	A	A	A	A	A	A
B	B	B	B	A	A	A	A	A	A
A	A	B	B	A	A	B	B	B	B
B	B	A	A	A	A	B	B	B	B
A	A	B	B	A	A	A	A	B	B
A	A	A	A	A	A	A	A	B	B
A	A	B	B	A	B	A	A	B	B
B	B	B	B	B	B	A	A	A	B
B	B	A	A	B	B	A	A	A	A
A	B	A	A	A	A	A	A	A	A
A	A	B	B	B	B	B	B	B	B
B	B	B	B	B	B	B	B	A	A
A	A	B	B	B	B	B	B	B	B
A	A	A	A	B	B	B	B	B	B
A	A	A	A	B	B	B	B	B	B
B	B	A	A	B	B	B	B	A	B
B	B	A	A	B	B	B	B	B	B
B	B	B	B	B	B	B	B	B	B
B	B	B	B	B	B	B	B	B	B

Table S3 Assay 2
A28; wt vs kd; wt=A, kd=B

rep1.F	rep1.M	rep2.F	rep2.M	rep3.F	rep3.M	rep4.F	rep4.M	rep5.F	rep5.M
B	B	B	B	A	A	A	A	B	B
B	B	A	A	A	A	A	A	B	B
B	B	A	B	B	B	A	B	A	A
A	B	A	A	B	B	B	B	A	A
A	A	A	A	B	B	A	A	A	A
B	B	B	B	B	B	B	B	A	A
B	B	B	B	B	B	B	B	A	A
B	B	B	B	B	B	B	B	A	A
A	B	B	B	B	B	A	B	B	B
A	A	A	A	B	A	A	B	B	B
A	A	A	A	B	B	B	B	B	B
A	A	B	B	B	B	A	A	A	A
B	B	B	B	B	B	A	A	B	B
B	B	B	B	A	A	B	B	B	B
A	A	A	A	A	A	B	B	A	A
A	A	B	A	B	B	B	A	A	A
A	A	B	A	A	A	A	A	B	B
A	A	A	A	A	A	A	A	B	B
B	B	A	A	A	A	A	A	B	B
B	B	A	A	A	B	A	A	B	B
B	B	B	B	A	A	B	B	A	B
A	A	B	B	A	A	B	B	A	A
A	A	A	A	A	A	B	B	B	B
B	A	A	A	A	A	B	A	A	A

Table S3 Assay 3
A28; wt vs kd; wt=A, kd=B

rep1.F	rep1.M	rep2.F	rep2.M	rep3.F	rep3.M	rep4.F	rep4.M	rep5.F	rep5.M
B	A	B	A	A	B	B	B	A	A
A	B	A	A	A	B	B	B	B	B
B	A	A	B	A	A	A	A	B	A
A	B	B	B	A	A	A	A	B	B
A	A	B	B	B	A	A	B	B	A
B	B	B	B	A	B	A	B	A	B
A	A	A	B	B	B	B	B	A	B
A	B	A	B	B	B	B	B	B	B
A	B	A	A	A	B	A	A	A	A
B	B	A	A	B	A	A	A	B	A
B	B	B	A	B	B	B	B	A	B
A	A	B	A	A	A	A	A	A	A
B	A	A	A	B	A	B	A	A	A
A	B	A	A	A	B	B	A	B	A
B	A	B	A	B	A	B	A	A	A
A	A	B	A	A	B	B	A	B	B
B	B	B	B	A	A	A	B	A	A
B	B	B	A	B	B	A	B	B	B
B	A	B	B	A	A	B	B	B	B
B	A	B	B	A	A	A	A	A	A
A	A	A	B	B	B	B	A	B	B
A	A	A	B	B	B	A	B	A	B
B	B	A	A	B	B	B	B	B	B
A	A	A	A	B	B	A	B	A	B

Table S3 Assay 4;
O11xO11; wt vs kd; wt=A, kd=B;

rep1.F	rep1.M	rep2.F	rep2.M	rep3.F	rep3.M	rep4.F	rep4.M	rep5.F	rep5.M
A	A	A	A	B	A	B	B	B	B
A	A	A	A	B	B	B	B	B	B
A	A	A	A	B	B	B	B	A	A
A	A	B	B	B	B	A	A	A	A
A	A	B	B	B	B	A	A	A	A
A	A	A	A	B	B	B	B	A	A
A	A	B	B	B	B	B	B	B	B
B	B	A	A	B	B	B	B	B	B
B	B	A	A	B	B	B	B	B	A
B	B	B	B	B	B	B	B	A	A
B	B	B	B	A	B	B	B	A	A
A	A	B	B	A	A	B	B	A	A
B	B	B	B	B	B	B	B	B	B
B	B	B	B	B	A	B	B	B	B
B	B	A	A	A	A	A	A	B	B
B	B	B	B	A	A	A	A	A	A
A	A	B	B	A	A	A	A	B	B
A	A	B	B	A	A	A	A	B	B
B	B	A	A	A	A	A	A	B	B
A	A	A	A	A	A	A	A	A	A
A	A	A	A	A	A	A	A	A	A
B	B	A	A	A	A	A	A	A	A
B	B	B	A	A	A	A	A	B	B
B	B	A	A	A	A	A	A	A	A

Table S3 Assay 5;
O11xO11; wt vs kd; wt=A, kd=B;

rep1.F	rep1.M	rep2.F	rep2.M	rep3.F	rep3.M	rep4.F	rep4.M	rep5.F	rep5.M
A	A	A	A	A	A	A	B	B	B
A	A	B	A	A	A	B	B	B	B
B	A	B	B	B	B	B	B	A	A
B	B	B	B	B	B	B	B	B	B
B	B	B	B	A	B	A	A	B	B
A	A	A	A	A	B	A	A	B	B
A	A	A	A	B	B	A	A	B	B
B	B	B	B	A	A	B	B	A	A
B	B	A	A	B	B	A	A	A	A
B	A	A	A	A	A	A	A	B	B
A	A	B	A	A	A	A	A	B	B
A	A	B	B	B	B	A	B	A	B
B	B	A	A	A	A	A	A	A	A
B	B	B	B	A	A	A	A	A	A
A	A	B	A	A	A	B	B	A	A
A	A	A	A	B	B	B	B	A	A
A	A	A	A	B	B	A	A	A	A
B	A	A	A	A	A	B	B	B	A
A	A	B	B	B	B	B	A	A	A
B	B	B	B	B	A	B	B	A	A
A	A	A	A	A	B	B	B	A	A
B	B	A	A	B	B	A	B	B	B
A	A	B	B	B	A	B	A	B	B
B	B	A	A	B	B	B	B	B	B

Table S3 Assay 6;
O11xO11; wt vs kd; wt=A, kd=B;

rep1.F	rep1.M	rep2.F	rep2.M	rep3.F	rep3.M	rep4.F	rep4.M	rep5.F	rep5.M
A	B	B	A	A	B	A	B	B	A
A	A	A	B	A	B	A	B	B	A
A	B	B	A	A	B	A	B	A	B
A	A	A	B	A	B	A	B	A	B
A	B	A	B	B	B	A	B	B	B
A	A	B	A	B	B	A	B	B	B
A	A	B	B	A	A	A	A	A	A
A	A	B	B	A	A	A	A	A	A
A	B	B	B	B	A	B	B	A	A
A	B	A	A	B	A	B	B	A	B
B	B	A	A	A	B	B	B	A	B
B	B	A	A	B	A	B	B	B	B
B	B	B	B	B	B	B	B	A	A
B	A	B	B	B	B	B	B	A	A
B	A	A	B	A	A	A	A	B	B
B	A	B	A	A	A	B	B	A	B
A	A	A	B	A	B	B	A	A	B
A	B	B	A	A	B	B	A	B	A
B	B	A	B	B	A	B	A	A	B
B	A	A	B	B	A	A	B	B	B
B	A	B	A	A	B	A	A	B	B
B	B	A	B	B	A	A	A	B	A
B	B	B	A	B	A	B	A	B	B
B	B	A	B	B	A	B	A	B	B

Table S3 Assay 07
isoA28; kd vs kd; 3 vs. 4; 3=A; 4=B

rep1.F	rep1.M	rep2.F	rep2.M	rep3.F	rep3.M	rep4.F	rep4.M	rep5.F	rep5.M
A	A	B	B	B	B	A	A	A	A
A	A	B	B	B	A	B	A	B	B
A	A	B	B	A	A	B	A	A	B
A	A	A	A	A	B	B	A	B	A
A	A	A	A	A	A	A	A	B	B
B	A	A	A	A	A	B	B	A	A
A	A	A	A	B	A	B	B	A	A
A	A	B	B	A	B	B	B	A	A
B	A	A	A	B	B	A	A	A	A
B	B	B	B	A	A	A	A	A	A
B	B	B	B	B	B	A	B	B	B
B	B	A	A	B	B	A	B	B	B
A	B	A	B	A	B	A	B	B	B
A	B	A	A	A	A	A	A	A	A
B	B	B	B	A	B	A	B	B	B
B	B	A	A	A	A	B	B	B	B
B	B	A	A	B	B	A	A	A	A
A	A	B	B	B	B	B	A	B	B
B	A	B	B	B	B	B	A	B	B
B	B	A	A	B	A	A	B	B	B
A	B	B	B	A	A	A	B	B	B
B	A	A	A	A	B	B	B	A	A
A	A	B	B	B	A	B	B	A	A
B	B	B	B	B	B	B	B	A	A

Table S3 Assay 08;
isoO11xisoO11;kd vs kd 1 vs 4 1=A, 4=B

rep1.F	rep1.M	rep2.F	rep2.M	rep3.F	rep3.M	rep4.F	rep4.M	rep5.F	rep5.M
B	A	A	B	A	A	B	B	A	A
B	A	A	B	A	A	B	B	B	B
A	B	A	A	A	A	B	B	A	A
A	B	A	A	A	A	A	A	A	A
A	A	B	B	A	A	A	A	A	A
B	B	B	B	A	A	A	A	B	A
B	A	B	B	A	A	A	A	A	B
A	B	B	B	A	A	A	B	B	A
B	B	B	B	A	A	B	A	B	B
B	B	A	A	A	A	A	A	A	A
B	B	A	A	B	B	A	A	B	B
A	A	A	A	B	B	A	A	B	B
A	A	B	B	B	A	A	A	A	A
A	A	B	B	B	A	A	A	B	B
A	A	B	B	A	A	A	A	A	A
B	B	B	B	A	A	B	B	B	B
A	A	B	A	B	B	B	B	B	B
A	B	B	A	B	B	B	B	A	A
A	B	A	A	B	B	A	A	A	A
B	A	B	B	B	B	B	B	A	B
B	B	A	A	B	B	B	B	A	B
A	B	A	B	B	A	B	B	B	A
B	B	A	B	B	B	B	A	B	A
B	B	A	A	B	B	B	A	B	A

Table S4 Assay 1
isoA28; wt vs gfr; wt=A gfr=B

rep1.F	rep1.M	rep2.F	rep2.M	rep3.F	rep3.M	rep4.F	rep4.M	rep5.F	rep5.M
B	B	A	A	A	A	B	B	A	A
B	B	B	B	A	A	B	B	A	A
B	B	B	B	A	A	B	B	A	A
A	A	B	B	B	B	A	A	B	B
A	A	B	B	B	B	A	A	B	B
A	A	B	B	B	B	A	A	A	A
A	B	A	A	B	B	A	A	A	A
A	A	A	A	B	B	B	B	A	A
A	A	A	A	B	B	B	B	A	A
B	B	A	B	A	A	A	A	B	B
B	B	A	A	A	A	A	A	B	B
B	B	A	A	A	A	A	A	B	B
A	A	A	A	B	B	B	B	A	A
A	A	A	A	A	A	B	A	A	A
A	A	A	B	A	A	A	A	A	A
B	B	B	B	A	A	A	A	B	B
B	B	B	B	B	B	B	B	A	A
A	A	A	A	B	B	B	B	B	B
A	A	B	B	B	B	A	B	B	B
B	B	A	A	B	B	B	B	A	A
B	B	B	B	B	A	A	A	B	B
B	B	B	B	A	A	B	B	B	B
A	A	B	B	A	B	A	A	B	B
B	B	B	B	A	A	B	B	B	B

AA 55
AB 5
BA 2
BB 58

Table S4 Assay 2
O11xO11; wt vs gfr wt=A, gfr=B

rep1.F	rep1.M	rep2.F	rep2.M	rep3.F	rep3.M	rep4.F	rep4.M	rep5.F	rep5.M
B	A	B	B	A	A	B	B	B	B
B	B	B	B	B	B	A	A	B	B
B	B	B	B	B	B	A	A	A	A
A	A	A	A	A	A	A	A	A	A
A	A	A	A	B	B	B	B	A	A
A	A	A	A	B	B	A	A	B	B
B	B	A	A	A	A	B	B	B	B
B	B	B	B	A	A	A	A	B	B
B	B	B	B	A	B	A	A	B	B
B	B	B	B	B	B	A	A	B	B
B	B	B	B	A	B	A	A	B	B
A	A	A	A	A	A	B	B	B	A
A	A	B	B	B	B	B	B	A	A
B	A	B	B	A	A	A	A	A	A
B	B	B	B	B	B	B	B	A	A
B	B	B	B	B	B	B	B	A	A
A	A	A	A	B	B	B	B	B	B
A	A	A	A	B	B	A	A	B	B
B	B	B	B	B	B	A	A	B	B
A	A	A	A	A	A	A	A	A	A
A	A	A	A	A	A	B	B	A	A
A	A	A	A	A	A	B	B	A	A
A	A	A	A	B	B	B	B	A	A
A	A	A	A	A	A	B	B	A	A

Table S4 Assay 3;
A28xA28; wt vs et; wt=A et=B;

rep1.F	rep1.M	rep2.F	rep2.M	rep3.F	rep3.M	rep4.F	rep4.M	rep5.F	rep5.M
A	A	A	A	A	A	A	A	A	A
A	A	A	B	A	A	A	A	A	B
A	B	A	B	B	B	A	A	B	A
A	B	A	A	B	A	B	B	B	B
A	A	A	A	A	B	A	A	A	B
A	A	A	B	A	A	A	A	B	B
A	B	B	B	B	A	A	A	A	B
A	B	B	B	B	B	A	B	A	B
A	A	A	B	A	B	A	B	B	B
A	B	B	B	A	A	B	B	B	B
A	B	B	A	B	A	A	A	B	A
A	B	B	A	A	A	B	B	A	A
B	A	B	B	A	A	B	B	A	A
B	B	B	B	A	A	B	B	B	B
B	A	A	B	A	A	A	B	A	B
B	B	B	A	A	A	A	B	A	A
B	A	A	A	B	A	B	B	A	A
B	B	B	B	B	B	A	B	A	A
B	A	B	A	B	B	B	B	B	A
B	B	B	A	B	B	B	B	A	B
B	A	A	A	A	A	B	A	B	A
B	B	A	B	B	A	B	A	B	A
B	A	B	A	B	B	B	B	B	B
B	A	A	B	B	B	B	B	B	B

Table S4 Assay 4;
O11xO11; wt vs et; wt=A et=B;

rep1.F	rep1.M	rep2.F	rep2.M	rep3.F	rep3.M	rep4.F	rep4.M	rep5.F	rep5.M
B	A	B	A	A	B	B	A	A	A
B	A	B	A	A	B	B	A	A	A
B	A	A	A	A	A	A	A	B	B
B	A	A	A	A	A	A	A	A	B
B	B	B	A	A	B	B	A	B	A
B	B	B	B	B	A	B	A	A	B
A	A	A	B	A	A	B	A	B	A
A	A	A	A	B	B	A	B	A	B
A	A	B	A	A	B	A	A	A	B
A	A	B	B	A	B	B	A	B	B
A	B	A	B	A	A	B	B	B	B
A	B	B	B	A	B	B	B	B	A
A	B	A	A	A	A	B	A	B	A
A	A	A	A	A	B	A	A	A	A
B	A	B	A	B	A	B	B	A	A
B	A	B	A	B	A	B	B	B	A
A	B	B	B	B	B	A	B	A	A
A	B	A	A	B	B	A	B	B	A
B	A	A	A	B	A	B	A	B	A
A	A	B	B	B	A	A	B	A	B
A	A	B	B	B	A	A	A	A	B
B	B	A	B	B	A	A	A	A	B
B	B	A	B	B	B	A	A	B	B
B	B	A	A	B	B	A	A	B	B

Table S4 Assay 5;
A28xA28; wt vs ps; wt=A ps=B;

rep1.F	rep1.M	rep2.F	rep2.M	rep3.F	rep3.M	rep4.F	rep4.M	rep5.F	rep5.M
A	A	A	A	A	A	A	B	A	A
A	A	A	A	A	A	A	B	B	A
A	A	B	A	A	A	A	A	B	A
A	B	B	A	B	B	A	B	B	B
B	B	A	A	B	B	A	A	B	B
B	B	A	A	A	B	A	A	B	B
B	A	B	B	A	B	B	B	B	B
B	B	B	B	A	A	B	B	A	B
B	B	A	B	B	A	A	A	A	B
A	A	B	A	B	A	B	A	A	B
A	A	A	B	A	A	A	B	A	A
B	A	B	A	B	A	A	A	A	A
B	A	A	A	A	A	A	A	A	B
A	B	A	A	A	B	A	A	A	B
A	B	A	B	A	B	A	B	B	A
A	A	A	B	A	A	B	A	B	A
A	A	B	B	B	B	B	A	B	B
B	B	B	B	A	B	B	B	A	B
A	B	A	B	B	B	B	B	A	B
A	B	B	A	B	B	B	A	B	A
B	B	A	B	B	B	B	A	B	A
B	B	B	B	B	A	B	B	A	B
B	A	B	A	B	A	B	B	B	A
B	A	B	A	B	A	B	A	A	B

Table S4 Assay 6;
O11xO11; wt vs ps; wt=A ps=B;

rep1.F	rep1.M	rep2.F	rep2.M	rep3.F	rep3.M	rep4.F	rep4.M	rep5.F	rep5.M
B	A	B	B	A	B	A	A	B	A
B	B	A	A	A	A	B	A	B	B
A	B	A	A	A	A	B	B	A	A
A	A	A	A	B	A	B	B	A	A
A	A	A	B	A	A	A	B	B	A
A	A	B	B	A	B	A	A	B	B
A	B	B	B	A	A	A	A	B	A
B	B	A	B	A	B	A	A	B	B
B	B	A	A	A	A	A	B	B	A
A	B	A	A	A	A	A	B	B	B
A	B	A	A	A	A	A	A	B	B
B	B	B	A	A	B	A	A	B	A
A	B	A	A	B	B	A	B	A	A
A	A	A	A	A	A	A	A	A	A
A	A	B	A	B	A	A	A	A	B
A	A	A	A	B	A	B	A	A	B
B	A	B	A	B	A	B	A	B	A
B	A	A	B	B	A	B	B	B	A
A	A	B	B	B	B	B	B	A	B
B	A	B	B	B	B	B	B	A	A
B	A	B	B	B	B	B	A	A	B
B	B	B	A	B	A	B	A	A	A
B	A	B	A	B	A	B	A	A	B
B	B	B	B	B	A	B	B	A	B

Table S4 Assay 7;
A28xO11; wt vs et; wt=A et=B;

rep1.F	rep1.M	rep2.F	rep2.M	rep3.F	rep3.M	rep4.F	rep4.M	rep5.F	rep5.M
B	B	A	A	B	B	B	B	B	B
A	A	B	B	B	B	B	B	B	B
B	B	A	A	A	A	B	B	A	A
A	A	A	A	A	A	B	B	A	A
A	A	A	B	A	A	B	B	A	A
A	B	A	A	B	B	B	B	A	A
B	B	A	A	B	B	B	B	A	A
B	B	B	B	A	A	B	B	A	A
A	A	B	B	A	A	B	B	B	B
A	A	A	A	A	A	B	B	B	B
B	B	A	A	A	A	A	A	A	A
A	A	A	A	B	B	A	A	B	B
A	A	B	B	B	B	A	A	A	A
A	A	B	B	B	B	A	A	B	B
A	A	B	B	B	B	A	A	B	B
A	A	B	B	B	B	A	A	B	B
B	B	B	B	B	B	A	A	B	B
B	B	A	A	B	B	A	A	A	A
B	B	A	A	A	A	A	A	A	A
B	B	B	B	A	A	A	A	A	A
A	B	A	A	B	B	B	B	B	B
B	B	B	B	A	A	B	B	B	B
B	B	B	B	A	A	A	A	B	B
B	B	B	B	A	A	A	B	A	A

Supplementary methods:
Statistical model for SII estimation

The purpose of this statistical procedure is to compute an unbiased estimate of the Sexual Isolation Index (SII). This rate of assortative mating (preferential choice of a mating partner from the same population) is not trivial to calculate when the number of partners is finite and remating is limited, as the mating choice for the last females is constrained by the choices of previously-mated females.

Both male and female flies from two distinct origins (by convention, A and B) are placed in a mating arena, and the formation of couples is tracked for a specific duration (generally, up to the point where all flies have been involved in a mating event). In such a context, calculating the rate of preferential mating based on the frequency of each couple (i.e. comparing pairs A-A + B-B vs. A-B + B-A) relies on the hypothesis that one population does not tend to mate earlier than the other. Indeed, if all females prefer males from population A and, for some reason, females from population A are also faster to mate, then all first couples can be A-A and all following ones can be B-B without any "real" assortative mating. It is thus necessary to differentiate the mating order from a real assortative mating trend.

We propose a statistical model that accounts for such a situation, expressing the frequencies of various pairs as a function of the rate of assortative mating (SII) as well as male/female preferential mating biases. The next section details how to estimate these parameters by maximum likelihood, and checks the accuracy of the model.

Probabilistic model

Let's assume a perfect experimental design, with N males and N females from two populations ($4N$ individuals in the mating area). The experiment stops when $2N$ couples have been recorded. In this section, the possibility of male remating will be neglected.

Model parameters are defined in an even sex-ratio population, i.e. when there are as many males and females from both populations. In this even population, the probability that the next mating female comes from the A population is $p_{♀}$, and the probability that the next mating male comes from the A population is $p_{♂}$. Although symmetric in the model, both rates can be interpreted differently: $p_{♀}$ can indicate the trend for females A to mate earlier, while $p_{♂}$ can be the

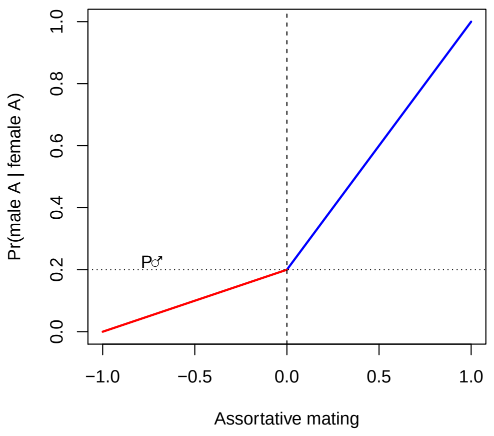

Figure 1: Conditional probability of picking a male A for a female A when the preference for male A differs from 1/2 (in this example, $p_{♂} = 0.2$). The model for positive assortative mating differs from negative assortative mating, justifying two distinct variants.

probability for a male A to be chosen first (preference shared by females from both populations). The rate of assortative mating h (equivalent to the sexual isolation index SII) measures the bias towards choosing a male from its own population. We will assume that the mating choice relies on females (males would mate equally with all females), and that choice probability scales linearly with frequencies (i.e. if the probability to choose a male from population B is p when the frequency of males from population B is f, the probability to chose a male from population B is $p/2$ when the frequency of B males is $f/2$).

The rate of assortative mating h can, in theory, vary between -1 (complete disassortative mating) to 1 (perfect assortative mating). However, positive and negative assortative mating are not symmetric, and both require a (slightly) different parameterization when $p_{♂} \neq 0.5$ (figure 1). The following will thus describe two distinct models for the cases $h \geq 0$ (positive assortative mating) and $h \leq 0$ (negative assortative mating, or disassortative mating).

Positive assortative mating ($0 \leq h \leq 1$)

In an even population, the probability that the first female is A is $p_♀$, and the probability that the first male is A is defined conditional to the population of the first female:

$$\begin{aligned}
\Pr(♀_A) &= p_♀ \\
\Pr(♀_B) &= 1 - p_♀ \\
\Pr(♂_A | ♀_A) &= h + p_♂(1-h) \\
\Pr(♂_B | ♀_A) &= 1 - h - p_♂(1-h) \\
\Pr(♂_A | ♀_B) &= p_♂(1-h) \\
\Pr(♂_B | ♀_B) &= 1 - p_♂(1-h).
\end{aligned}$$

When the proportions of males and females from populations A and B are not even, these probabilities have to be modified. Noting $f_♀$ and $f_♂$ the proportion of females and males respectively from population A:

$$\begin{aligned}
\Pr(♀_A) &= \frac{p_♀ f_♀}{K_1} \\
\Pr(♀_B) &= \frac{(1-p_♀)(1-f_♀)}{K_1} \\
\Pr(♂_A | ♀_A) &= \frac{f_♂(h + p_♂(1-h))}{K_2} \\
\Pr(♂_B | ♀_A) &= \frac{(1-f_♂)(1-p_♂)(1-h)}{K_2} \\
\Pr(♂_A | ♀_B) &= \frac{f_♂ p_♂(1-h)}{K_3} \\
\Pr(♂_B | ♀_B) &= \frac{(1-f_♂)(1-p_♂(1-h))}{K_3}
\end{aligned}$$

with:

$$\begin{aligned}
K_1 &= p_♀ f_♀ + (1-p_♀)(1-f_♀), \\
K_2 &= 1 - f_♂ + (h + p_♂(1-h))(2f_♂ - 1), \\
K_3 &= 1 - f_♂ + p_♂(1-h)(2f_♂ - 1).
\end{aligned}$$

The probabilities to observe each of the four possible couples are thus:

$$\begin{aligned}
\Pr(\female_A \male_A) &= \frac{1}{K_1 K_2} p_\female f_\female f_\male (h + p_\male (1-h)), \\
\Pr(\female_A \male_B) &= \frac{1}{K_1 K_2} p_\female f_\female (1 - p_\male)(1 - f_\male)(1-h), \\
\Pr(\female_B \male_A) &= \frac{1}{K_1 K_3} (1 - p_\female)(1 - f_\female) f_\male p_\male (1-h), \\
\Pr(\female_B \male_B) &= \frac{1}{K_1 K_3} (1 - p_\female)(1 - f_\female)(1 - f_\male)(1 - p_\male (1-h)).
\end{aligned}$$

Disassortative mating ($-1 \leq h \leq 0$)

The reasoning is similar to the positive assortative mating case:

$$\begin{aligned}
\Pr(\female_A) &= p_\female \\
\Pr(\female_B) &= 1 - p_\female \\
\Pr(\male_A | \female_A) &= p_\male (1+h) \\
\Pr(\male_B | \female_A) &= 1 - p_\male (1+h) \\
\Pr(\male_A | \female_B) &= p_\male (1+h) - h \\
\Pr(\male_B | \female_B) &= 1 - p_\male (1+h) + h.
\end{aligned}$$

Accounting for uneven frequencies:

$$\begin{aligned}
\Pr(\female_A) &= \frac{p_\female f_\female}{K_1} \\
\Pr(\female_B) &= \frac{(1 - p_\female)(1 - f_\female)}{K_1} \\
\Pr(\male_A | \female_A) &= \frac{f_\male p_\male (h+1)}{K_4} \\
\Pr(\male_B | \female_A) &= \frac{(1 - f_\male)(1 - p_\male)(1+h)}{K_4} \\
\Pr(\male_A | \female_B) &= \frac{f_\male (p_\male (1+h) - h)}{K_5} \\
\Pr(\male_B | \female_B) &= \frac{(1 - f_\male)(1 + h - p_\male (1+h))}{K_5}
\end{aligned}$$

with:

$$K_4 = (1 - f_{\male})(1 - p_{\male}(1 + h)) + f_{\male} p_{\male}(1 + h),$$
$$K_5 = f_{\male}(p_{\male}(1 + h) - h) + (1 - f_{\male})(1 + h - p_{\male}(1 + h)).$$

Finally:

$$\Pr(\female_A \male_A) = \frac{1}{K_1 K_4} p_{\female} f_{\female} f_{\male} p_{\male}(1 + h),$$
$$\Pr(\female_A \male_B) = \frac{1}{K_1 K_4} p_{\female} f_{\female}(1 - f_{\male})(1 - (1 + h)p_{\male}),$$
$$\Pr(\female_B \male_A) = \frac{1}{K_1 K_5}(1 - p_{\female})(1 - f_{\female}) f_{\male}(p_{\male}(1 + h) - h),$$
$$\Pr(\female_B \male_B) = \frac{1}{K_1 K_5}(1 - p_{\female})(1 - f_{\female})(1 - f_{\male})(1 + h - p_{\male}(1 + h)).$$

Remating

While females generally wait for hours or days between mating events, it is not impossible that some males could mate several times during the experiment. The possibility of remating is taken into account by modifying the frequency of individuals from each population. The remating rate r is between 0 and 1, $r = 0$ stands for no remating, while $r = 1$ means that the mating process does not depend on the frequencies (all individuals are available for mating). In practice, the following frequencies were used in the above equations:

$$f'_{\male} = f_{\male} - r(f_{\male} - \frac{1}{2}),$$

so that $r > 0$ is equivalent to a frequency closer to $1/2$.

The rate of remating is rarely of interest, and may not affect substantially other parameters. However, it is necessary to take it into account to get a non-null likelihood in cases that can be explained only by one or a few remating events.

Statistical framework

A dataset consists in a series of observed successive pairs during an experiment, i.e. an ordered set of Z females $\boldsymbol{F} = (F_1, F_2, \ldots, F_Z)$ and Z males $\boldsymbol{M} = (M_1, \ldots, M_Z)$. Only two populations were compared, and all Fj and M_j can take only two states, "A" or "B". In absence of remating and if all individuals were involved in pairs, the expectation is that $Z = 2N$. In practice, the real number of observations can deviate from this expectation due to male remating.

Experiments were replicated n times, and we will denote $\boldsymbol{F}_i$ $(1 \leq i \leq n)$ the ith replicate (and thus, F_{ij} stands for the population of the jth mating female in the ith replicate).

The probability to observe a pair $\Pr(F_{ij}, M_{ij})$ can be calculated as stated above as a function of four parameters: $p_{♀}$, $p_{♂}$, h, and r. Frequencies of remaining females and males $f_{♀}$ and $f_{♂}$ are determined from the dataset. The probability to observe a full time series is:

$$\Pr(\boldsymbol{F}_i, \boldsymbol{M}_i) = \prod_{j=1}^{Z} \Pr(F_{ij}, M_{ij}),$$

and the probability to observe the full experiment $\boldsymbol{D} = (\boldsymbol{F}_1, \boldsymbol{M}_1, \boldsymbol{F}_2, \boldsymbol{M}_2, \ldots, \boldsymbol{F}_n, \boldsymbol{M}_n)$ was calculated assuming independent replicates:

$$\Pr(\boldsymbol{D}) = \prod_{i=1}^{n} \Pr(\boldsymbol{F}_i, \boldsymbol{M}_i).$$

Parameter estimation was performed by maximizing numerically the likelihood function

$$L(p_{♀}, p_{♂}, h, r | \boldsymbol{D}) = \Pr(\boldsymbol{D}),$$

with the `mle` function (package `stats4`) in `R` version 3.3.1, and confidence intervals were obtained by profiling the likelihood function (scripts available as supplementary material).

Three simpler models were also fitted (without assortative mating, without female mating bias, and without male mating bias, respectively):

$$\begin{aligned} L^h(p_{♀}, p_{♂}, r | \boldsymbol{D}, h = 0) &= \Pr(\boldsymbol{D}), \\ L^{p_{♀}}(p_{♂}, h, r | \boldsymbol{D}, p_{♀} = \frac{1}{2}) &= \Pr(\boldsymbol{D}), \\ L^{p_{♂}}(p_{♀}, h, r | \boldsymbol{D}, p_{♂} = \frac{1}{2}) &= \Pr(\boldsymbol{D}). \end{aligned}$$

Model comparison was based on classical likelihood ratio tests, assuming that the "simple" models represent null hypotheses to test:

$$\begin{aligned} \alpha^h &= -2 \log \frac{\hat{L}^h}{\hat{L}}, \\ \alpha^{p_{♀}} &= -2 \log \frac{\hat{L}^{p_{♀}}}{\hat{L}}, \\ \alpha^{p_{♂}} &= -2 \log \frac{\hat{L}^{p_{♂}}}{\hat{L}}, \end{aligned}$$

where $\hat{L}$ is the maximum of the likelihood function. Approximate P-values were calculated based on the assumption that αs follow χ^2 distributions with one degree of freedom.

Tests

Simulations were run to assess the properties of the statistical model. First, we tried to determine whether the model was able to estimate the assortative mating index h from realistic datasets. Simulated vs. estimated values of h are indicated in fig 2, showing a good agreement. The error in the estimates is higher when there is only one replicate ($n = 1$), but estimates are not biased.

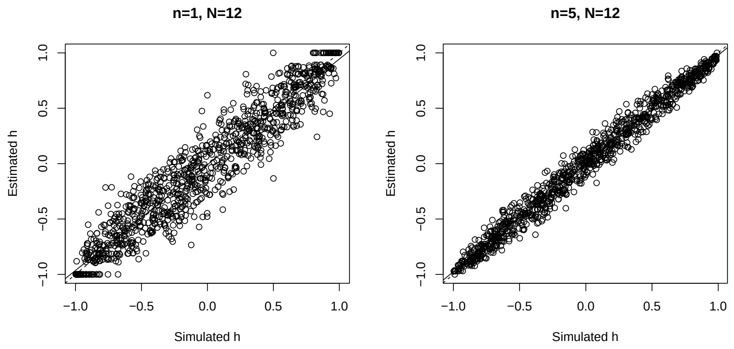

Figure 2: Accuracy of the maximum-likelihood estimates of the rate of assortative mating.

A power analysis lead to convincing results (Table 1). For low sample sizes ($n = 1$ replicates), the likelihood ratio test tends to be conservative (rejection rate of H_0 lower than the theoretical P-value$= 0.05$ threshold). Assortative mating rates of the order of magnitude of $h = 0.1$ are nearly impossible to detect while rates of $h = 0.5$ are detected in the majority of data sets. The other model parameters ($p_{♂}$, $p_{♀}$, and r) do not affect the power of the test (at least in the tested conditions). Including five replicates increases the power to detect assortative mating substantially, although it is still limited for $h = 0.1$.

As expected, the Fisher Exact test performs well when both populations mate at the same rate, but the false positive rate increases substantially when both $p_{♂}$ and $p_{♀}$ deviate from $1/2$. Surprisingly, this test also lacks power compared with the maximum likelihood model, justifying the use of our more complex model even in absence of mating order issues.

Table 1: Power of the Likelihood-ratio test on unique replicated simulated datasets ($n = 1, N = 12$, top) and on five-replicate datasets ($n = 5, N = 12$, bottom). The table indicates the frequency (out of 1000 replicates) of rejection of the null hypothesis H_0: $h = 0$ for a P-value threshold of 0.05. Note the inflated risk of false positives (bold faced) with the homogeneity test when both $p_♂$ and $p_♀$ differ from 1/2.

$n = 1$

$p_♂$	$p_♀$	r	$h = -0.1$	$h = 0$	$h = 0.1$	$h = 0.5$	$h = 0.8$
0.50	0.50	0.00	0.06	0.06	0.07	0.66	0.99
0.75	0.50	0.00	0.07	0.04	0.07	0.70	0.99
0.75	0.75	0.00	0.07	0.04	0.08	0.68	0.98
0.50	0.50	0.20	0.07	0.06	0.09	0.68	1.00

$n = 5$

$p_♂$	$p_♀$	r	$h = -0.1$	$h = 0$	$h = 0.1$	$h = 0.5$	$h = 0.8$
0.50	0.50	0.00	0.16	0.05	0.17	1.00	1.00
0.75	0.50	0.00	0.18	0.04	0.20	1.00	1.00
0.75	0.75	0.00	0.13	0.06	0.28	1.00	1.00
0.50	0.50	0.20	0.21	0.06	0.21	1.00	1.00

Fisher $n = 1$

$p_♂$	$p_♀$	r	$h = -0.1$	$h = 0$	$h = 0.1$	$h = 0.5$	$h = 0.8$
0.50	0.50	0.00	0.06	0.04	0.05	0.61	0.99
0.75	0.50	0.00	0.06	0.04	0.06	0.62	0.98
0.75	0.75	0.00	0.06	**0.15**	0.25	0.83	1.00
0.50	0.50	0.20	0.05	0.03	0.05	0.59	0.99

Fisher $n = 5$

$p_♂$	$p_♀$	r	$h = -0.1$	$h = 0$	$h = 0.1$	$h = 0.5$	$h = 0.8$
0.50	0.50	0.00	0.16	0.05	0.17	1.00	1.00
0.75	0.50	0.00	0.18	0.05	0.17	1.00	1.00
0.75	0.75	0.00	0.17	**0.59**	0.87	1.00	1.00
0.50	0.50	0.20	0.15	0.04	0.15	1.00	1.00
